# Lifetime reproductive output: individual stochasticity, variance, and sensitivity analysis

**DOI:** 10.1007/s12080-017-0335-2

**Published:** 2017-04-17

**Authors:** Silke F. van Daalen, Hal Caswell

**Affiliations:** Institute for Biodiversity and Ecosystem Dynamics, University of Amsterdam, PO Box 94248, 1090 GE Amsterdam, The Netherlands

**Keywords:** Lifetime reproductive output, Matrix population models, Individual stochasticity, Sensitivity analysis, Markov chains with rewards, Opportunity for selection, Inter-individual variance

## Abstract

Lifetime reproductive output (LRO) determines per-generation growth rates, establishes criteria for population growth or decline, and is an important component of fitness. Empirical measurements of LRO reveal high variance among individuals. This variance may result from genuine heterogeneity in individual properties, or from individual stochasticity, the outcome of probabilistic demographic events during the life cycle. To evaluate the extent of individual stochasticity requires the calculation of the statistics of LRO from a demographic model. Mean LRO is routinely calculated (as the net reproductive rate), but the calculation of variances has only recently received attention. Here, we present a complete, exact, analytical, closed-form solution for all the moments of LRO, for age- and stage-classified populations. Previous studies have relied on simulation, iterative solutions, or closed-form analytical solutions that capture only part of the sources of variance. We also present the sensitivity and elasticity of all of the statistics of LRO to parameters defining survival, stage transitions, and (st)age-specific fertility. Selection can operate on variance in LRO only if the variance results from genetic heterogeneity. The potential opportunity for selection is quantified by Crow’s index $\mathcal {I}$, the ratio of the variance to the square of the mean. But variance due to individual stochasticity is only an *apparent* opportunity for selection. In a comparison of a range of age-classified models for human populations, we find that proportional increases in mortality have very small effects on the mean and variance of LRO, but large positive effects on $\mathcal {I}$. Proportional increases in fertility increase both the mean and variance of LRO, but reduce $\mathcal {I}$. For a size-classified tree population, the elasticity of both mean and variance of LRO to stage-specific mortality are negative; the elasticities to stage-specific fertility are positive.

## Introduction

Like all men in Babylon, I have been proconsul; like all, I have been a slave. I have known omnipotence, ignominy, imprisonment … I owe this almost atrocious variety to an institution which other republics know nothing about, and which operates among them imperfectly and in secret: the lottery. Jorge Luis Borges, *The Lottery in Babylon*

Lifetime reproductive output (LRO) is, as the name implies, the total production of offspring over the lifetime of an individual^1^ and is one of the most important characteristics of an individual life history. The expectation of LRO, calculated in terms of female offspring per female, is the net reproductive rate *R*_0_. In ecology, the critical value *R*_0_ = 1 defines the boundary separating population growth and persistence from population decline and extinction. In epidemiology, *R*_0_ for a pathogen determines whether a disease will or will not cause an outbreak. In evolutionary biology, *R*_0_ is a critical component of fitness (sometimes considered to *be* fitness, although that is sometimes problematic).

Genetic variance in LRO is considered to be the raw material on which natural selection operates. Crow (1958) introduced an index, which now bears his name, of the “opportunity for selection.” If *X* denotes some measure of fitness, then the opportunity for selection is

1 $$ \mathcal{I} = \frac{V(X)}{E(X)^2} , $$

also known as the standardized variance. It gives the maximum rate of evolutionary change that could be produced by selection if all the variance in *X* were genetic. To rigorously investigate opportunities for selection would require more explicit population genetic models. Even so, the opportunity for selection is widely used in studies of both animal and human populations (e.g., Jones 2009; Brown et al. 2009; Robbins et al. 2011; Moorad et al. 2011; Courtiol et al. 2012).

Empirical studies of individual LRO routinely find large variance and usually a positive skew. Most individuals produce few, or no, offspring, while a few rare individuals produce many offspring (Clutton-Brock 1988; Newton 1989). These differences in LRO have two possible sources. One is heterogeneity—differences in the properties of individuals—including genetic heterogeneity, physiological differences, phenotypic plasticity, and environmental heterogeneity.

However, the differences may also be due to individual stochasticity (Caswell 2009, 2011, 2014; van Daalen and Caswell 2015). Individual stochasticity refers to differences among individuals due to the accumulation of random outcomes of the stochastic processes of mortality, growth, development, breeding, etc. Individual stochasticity would lead to variance among individuals even if they were totally identical and experienced exactly the same demographic rates.^2^ Depending on the outcome under consideration, variance due to individual stochasticity can be as great as or even exceed that caused by unobserved heterogeneity (Tuljapurkar et al. 2009; Steiner et al. 2010; Caswell 2011, 2014; Hartemink et al. 2017). Thus, before invoking heterogeneity as the explanation for differences among individuals, it is important to calculate the variation due to stochasticity as a kind of “neutral model” for variation (Steiner and Tuljapurkar 2012).

Individual stochasticity itself has two components. Consider an individual at some stage in its life cycle (say, at birth). The growth, development, and eventual death of this individual define a path through the stages of the life cycle. The pathways of two or more identical individuals, subject to the same rates at every stage of the life cycle, will differ randomly among themselves, and this variation among pathways is one source of individual stochasticity. At each step along an individual’s path, it may or may not reproduce. If it reproduces, the number of its offspring will be drawn from some probability distribution. This within-pathway stochastic variation in (stage-specific) fertility is the second source of individual stochasticity.

Our goal is to calculate the variance (and other statistics) of LRO, due to individual stochasticity, from basic demographic information, just as *R*_0_ can be calculated from age-classified or stage-classified demographic models (Rhodes 1940; Cushing and Zhou 1994; De Camino-Beck and Lewis 2007; Cushing and Ackleh 2012). To calculate the variance in LRO, we need to account for the generally infinite number of pathways through the life cycle, calculate the probabilities of each path, calculate the distribution of reproductive output at each stage on each path, and then integrate those probabilities to calculate the mean, variance, etc. of LRO.

The calculation of variance in LRO has been approached in several ways. Early studies used simulation to generate random trajectories through stages, including stages defined by reproductive output, to create a sample of lives from which variance in LRO could be calculated (e.g., Tuljapurkar et al. 2009; Steiner et al. 2010). An analytical solution for all the moments of LRO was provided by Caswell (2011) and provides the framework for our results here. That result took the form of an iterative calculation rather than a closed form expression. Steiner and Tuljapurkar (2012) presented a closed form analytical solution for all the moments of one component of LRO, using a moment generating function approach. They also reported simulations of the complete distribution of LRO and explored effects of heterogeneity. However, their solution included only part of the variance in LRO. They assumed the fertility of each age or stage to be a fixed deterministic quantity, neglecting the within-pathway component of variance. For example, the age-specific fertility of a 26-year-old Japanese woman in 1950 was 0.25. The analysis of Steiner and Tuljapurkar (2012) assumes that every 26-year-old woman, without exception, produces one fourth of a baby. In our framework, every 26-year-old woman, without exception, produces one baby with a probability of 0.25 and zero babies with a probability of 0.75 (Caswell 2011). This component of variance is biologically realistic and quantitively important. For example, analysis of 40 developed countries during the second demographic transition found that, as life expectancy increased, the fraction of variance in LRO due to within-trajectory randomness increased from ∼ 50*%* to ∼ 99*%* (Van Daalen and Caswell 2015). We show further examples and present the methodology for decomposing variance into these components, below.

All the studies published so far agree in finding that the variance in LRO due to individual stochasticity can comprise a high proportion of the observed variance in LRO, and that to ignore it is to miss a major source of variation (e.g., Tuljapurkar et al. 2009; Steiner et al. 2010; Steiner and Tuljapurkar 2012; Caswell 2011; van Daalen and Caswell 2015).

In this paper, we present exact, closed form, analytical formulae for all the moments of LRO, extending and replacing the iterative formulae of Caswell (2011). We include both the between-trajectory and within-trajectory components of variance and incorporate variance in stage-specific fertility either empirically or by a statistical model. We also present the sensitivity and elasticity analysis of means, variances, and all moments of LRO due to individual stochasticity. Our results can be applied to any age- or stage-classified matrix population model and to constant, periodic, or stochastic environments.

Our results rely on a mathematical model called a Markov chain with rewards, which we describe in “Markov chains with rewards as a model for LRO.” The construction of a Markov chain with rewards requires demographic information on survival, stage transitions, and fertility. Section “The statistics of LRO” presents the calculation of all the moments and other descriptive statistics of LRO. Section “Sensitivity analysis of LRO” presents the sensitivity and elasticity analysis of LRO. Section “Examples” presents examples of age-classified and stage-classified populations, and “Discussion” concludes with a discussion of results and possible extensions. Proofs and derivations appear in Appendix A.

## Markov chains with rewards as a model for LRO

### Notation

Matrices are denoted by uppercase boldface letters (e.g., **P**), and vectors by lowercase boldface letters (e.g., ***ρ***). Vectors are column vectors by default; **X** ^T^ is the transpose of **X**. The vector **1**_*n*_ is a *n* × 1 vector of ones, **I**_*n*_ is the identity matrix of order *n*, and **e**_*i*_ is the *i*th unit vector, with a 1 in the *i*th entry and zeros elsewhere. The matrix **E** is a matrix of ones, and **E**_*ij*_ is a matrix with a 1 in the (*i*,*j*) entry and zeros elsewhere. The diagonal matrix with the vector **x** on the diagonal and zeros elsewhere is denoted $\mathcal {D}(\textbf {x})$. The symbol ∘ denotes the Hadamard, or element-by-element product, and ⊗ denotes the Kronecker product. The vec operator vec **X** stacks the columns of an *m* × *n* matrix **X** into an *m* *n* × 1 column vector. The vec-permutation matrix **K**_*m*,*n*_ satisfies vec **X** ^T^ = **K**_*m*,*n*_vec **X**. In cases where it will help understanding, we indicate the dimension of displayed matrix expressions.

### The life cycle as a Markov chain

Our approach uses a mathematical model called a Markov chain with rewards. These models have a long history in stochastic process theory (e.g., Howard 1960; Puterman 1994; Sheskin 2010) but have only recently been applied to study lifetime reproduction (Caswell 2011; Van Daalen and Caswell 2015).

The individual life cycle is described by a finite-state, discrete-time, absorbing Markov chain; absorbing states represent death. The population projection matrix is written

2 $$ \textbf{A} = \textbf{U} + \textbf{F} $$

where **U** contains the transition probabilities for extant individuals and **F** contains stage-specific fertilities. Let *τ* be the number of transient (living) states, *α* the number of absorbing states, and *s* = *τ* + *α* the total number of states. Then the Markov chain transition matrix, including both transient and absorbing states, can be written
3 where the dimensions of the submatrices are noted. The matrix **P** is column stochastic. We assume that the spectral radius of **U** is strictly less than 1; thus, any individual eventually dies with probability 1. The probability of dying while in each of the transient states is contained in the mortality matrix **M**.

#### Reproductive rewards

An individual experiences a sequence of transitions according to the probabilities in **P**. Associated with each transition (including the transition of remaining in a stage) is a “reward” representing, in our case, reproductive output. The reproductive reward is a random variable with a specified set of moments. Rewards accumulate over the lifetime of the individual; the total accumulation at the time of death is the LRO. We make the reasonable assumption that individuals in the absorbing state stop accumulating rewards.^3^

The reproductive rewards associated with each transition are given by a set of matrices **R**_*k*_, *k* = 1,2,…. The (*i*,*j*) entry of **R**_*k*_ is the *k*th moment of the reproductive output associated with the transition *j* → *i*; that is,

4 $$ \mathbf{R}_{k} = \left( \begin{array}{c} E \left[ r_{ij}^{k} \right] \end{array}\right) . $$

These matrices **R**_*m*_ can be obtained in several ways, discussed in detail in Caswell (2011).

Empirical distribution.: The moments can be calculated empirically from data on individual reproduction. Such data are frequently obtained, but typically only mean reproductive output is reported. In the absence of this information, the reward matrices can be modelled by any probability distribution that is determined by its mean, including the following.
Bernoulli distribution.: When only a single offspring is produced, mean offspring production equals the probability of reproducing. The matrices of second and third moments satisfy
  5 $$ \textbf{R}_{3} = \textbf{R}_{2} = \textbf{R}_{1}.  $$
Poisson distribution.: When multiple offspring are produced, the Poisson distribution describes a situation where every individual has the same chances of producing those offspring. The moments satisfy
  6 $$\begin{array}{@{}rcl@{}} \textbf{R}_{2} &=& \textbf{R}_{1} + \left( \textbf{R}_{1} \circ \textbf{R}_{1} \right) \end{array} $$
  7 $$\begin{array}{@{}rcl@{}} \textbf{R}_{3} &=& \textbf{R}_{1} + 3 \left( \textbf{R}_{1} \circ \textbf{R}_{1} \right) +\left( \textbf{R}_{1} \circ \textbf{R}_{1} \circ \textbf{R}_{1} \right). \end{array} $$
Fixed rewards.: It is possible to eliminate variance in fertility by creating reward matrices where every individual in a given stage produces exactly the mean number of offspring, in which case
  8 $$\begin{array}{@{}rcl@{}} \textbf{R}_{2} &=& \textbf{R}_{1} \circ \textbf{R}_{1} \end{array} $$
  9 $$\begin{array}{@{}rcl@{}} \textbf{R}_{3} &=& \textbf{R}_{1} \circ \textbf{R}_{1} \circ \textbf{R}_{1}. \end{array} $$

In most demographic models, mean fertility is stage-specific rather than transition-specific. That is, mean fertility is described by a vector **f** that typically appears in the first row of the matrix **A**. An individual in stage *i* produces, on average, *f*_*i*_ offspring per time step, regardless of what transition the individual may make. In this case
10

11 $$\begin{array}{@{}rcl@{}} {\kern15pt}=\mathbf{1}_{s} \left( \begin{array}{c|c} \textbf{f}^{\textsf{T}} & \textbf{0}_{1 \times \alpha} \end{array}\right) . \end{array} $$

If parental survival is required for reproduction, then the last row[s] of **R**_1_ should be set to zero.

### The statistics of LRO

In this model, every individual is subject to the same rates at any given stage, so there is no heterogeneity. Even so, each individual may experience a different life course. The resulting variation among individuals is due to the individual stochasticity implied by the vital rates in **P** and the fertility process described by the **R**_*i*_. Our task is to derive the statistics of LRO from this information.

Let ***ρ***_*k*_ be a vector (dimension *s* × 1) whose *i*th entry is the *k*th moment of the remaining lifetime reproductive output of an individual starting in state *i*, i.e.,

12 $$ \boldsymbol{\rho}_{k} = \left( \begin{array}{c} E \left[ {\rho_{i}^{k}} \right] \end{array}\right) . $$

Because we assume that the dead do not reproduce, we know that the entries of ***ρ***_*k*_ corresponding to absorbing states are zero. Thus, we obtain the complete statistics of LRO if we can find the moment vectors $\tilde {\boldsymbol {\rho }}_{k}$, where $\tilde {\boldsymbol {\rho }}$ contains the first *τ* entries of ***ρ***, corresponding to the living stages. This vector is given by

13 $$ \tilde{\boldsymbol{\rho}} = \textbf{Z} \boldsymbol{\rho} \qquad \text{and} \qquad \boldsymbol{\rho} = \textbf{Z}^{\textsf{T}} \tilde{\boldsymbol{\rho}} $$

where

14 $$ \textbf{Z} = \left( \begin{array}{c|c} \textbf{I}_{\tau \times \tau} & \textbf{0}_{\tau \times \alpha} \end{array}\right) ;  $$

in terms of this matrix, $\boldsymbol {\rho } = \textbf {Z}^{\textsf {T}} \tilde {\boldsymbol {\rho }}$. We also define $\tilde {\textbf {R}}_{k}$, the *τ* × *τ* submatrix of **R**_*k*_ corresponding to transitions among the transient states:

15 $$ \tilde{\textbf{R}}_{k} = \textbf{Z} \textbf{R}_{k} \textbf{Z}^{\textsf{T}} . $$

In terms of these quantities, the moments of LRO are given by the following theorem.

#### **Theorem 1**

*The moment vectors* $\tilde {\boldsymbol {\rho }}$ *of lifetime accumulated reproductive output are*

16 $$\begin{array}{@{}rcl@{}} \tilde{\boldsymbol{\rho}}_{1} &=& \text{\textbf{N}}^{\text{\textsf{T}}} \text{\textbf{Z}} \left( \text{\textbf{P}} \circ \text{\textbf{R}}_{1} \right)^{\text{\textsf{T}}} \mathbf{1}_{s} \end{array} $$

17 $$\begin{array}{@{}rcl@{}} \tilde{\boldsymbol{\rho}}_{2} &=& \text{\textbf{N}}^{\text{\textsf{T}}} \left[ \text{\textbf{Z}} \left( \text{\textbf{P}}\circ\text{\textbf{R}}_{2} \right)^{\text{\textsf{T}}} \mathbf{1}_{s} \,+\, 2 \left( \text{\textbf{U}} \circ \tilde{\text{\textbf{R}}}_{1} \right)^{\text{\textsf{T}}} \tilde{\boldsymbol{\rho}}_{1} \right] \end{array} $$

18 $$\begin{array}{@{}rcl@{}} \tilde{\boldsymbol{\rho}}_{3} &=& \text{\textbf{N}}^{\text{\textsf{T}}} \left[ \text{\textbf{Z}} \left( \text{\textbf{P}} \circ\text{\textbf{R}}_{3} \right)^{\text{\textsf{T}}} \mathbf{1}_{s} \,+\, 3 \left( \text{\textbf{U}} \circ \tilde{\text{\textbf{R}}}_{2} \right)^{\text{\textsf{T}}} \tilde{\boldsymbol{\rho}}_{1} \,+\, 3 \left( \text{\textbf{U}} \circ\tilde{\text{\textbf{R}}}_{1} \right)^{\text{\textsf{T}}} \tilde{\boldsymbol{\rho}}_{2} \right] \end{array} $$

and, in general,

19 $$ \tilde{\boldsymbol{\rho}}_{m} =\text{\textbf{N}}^{\text{\textsf{T}}}\text{\textbf{Z}} \left( \text{\textbf{P}} \circ\text{\textbf{R}}_{m} \right)^{\text{\textsf{T}}} \mathbf{1}_{s} + \sum\limits_{k=1}^{m-1} \binom{m}{k}\text{\textbf{N}}^{\text{\textsf{T}}} \left( \text{\textbf{U}} \circ \tilde{\text{\textbf{R}}}_{m-k} \right)^{\text{\textsf{T}}} \tilde{\boldsymbol{\rho}}_{k}  $$

where **N** =(**I**_*τ*_ −**U**)^−1^ is the fundamental matrix of the Markov chain. 

#### *Proof*

See Appendix A □

In terms of the moment vectors provided by Theorem 1, the mean, variance, standard deviation, and coefficient of variation of LRO, and Crow’s index $\mathcal {I}$ of opportunity for selection, are given by

20 $$\begin{array}{@{}rcl@{}} E(\tilde{\boldsymbol{\rho}}) &=& \tilde{\boldsymbol{\rho}}_{1} \end{array} $$

21 $$\begin{array}{@{}rcl@{}} V(\tilde{\boldsymbol{\rho}}) &=& \tilde{\boldsymbol{\rho}}_{2} - \left( \tilde{\boldsymbol{\rho}}_{1} \circ \tilde{\boldsymbol{\rho}}_{1} \right) \end{array} $$

22 $$\begin{array}{@{}rcl@{}} SD(\tilde{\boldsymbol{\rho}}) &=& \sqrt{V(\tilde{\boldsymbol{\rho}})} \end{array} $$

23 $$\begin{array}{@{}rcl@{}} CV(\tilde{\boldsymbol{\rho}}) &=& \mathcal{D}\left( {\boldsymbol{\rho}}_{1} \right)^{-1} SD(\tilde{\boldsymbol{\rho}}) \end{array} $$

24 $$\begin{array}{@{}rcl@{}} \mathcal{I} &=& CV(\tilde{\boldsymbol{\rho}}) \circ CV(\tilde{\boldsymbol{\rho}}). \end{array} $$

We focus in this paper on statistics obtained from the first and second moments; these statistics, such as the variance, describe variability of the distribution. Calculations of the skewness, as a measure of the shape of the distribution, are given in Caswell (2011) and Van Daalen and Caswell (2015). All of the methods we present here can be applied to these statistics.

### Partitioning variance: within and between pathways

Variance in LRO is partly due to individuals following different pathways through the life cycle and partly due to variance in stage-specific fertility along those pathways. The overall variance $V (\tilde {\boldsymbol {\rho }})$ can be decomposed into these components using the law of conditional variance

25 $$ V(\tilde{\boldsymbol{\rho}}) = V_{\text{within}} + V_{\text{between}}  $$

(Rényi 1970, Theorem 1, p. 275). To calculate the variance between trajectories, we eliminate the within-trajectory variance by calculating $V(\tilde {\boldsymbol {\rho }})$ using the fixed reward model, in which the second moment matrix **R**_2_ is given by Eq. 8. The within-trajectory variance is obtained by subtraction as $V_{\text {within}}=V(\tilde {\boldsymbol {\rho }}) - V_{\text {between}}$.

## Sensitivity analysis of LRO

The statistics of LRO depend on the life history parameters that determine the transition matrices **U** and **M**, and the moment matrices **R**_1_, **R**_2_, …. Sensitivity analysis reveals how these parameters affect LRO; our goal is to derive the sensitivity and elasticity of LRO to changes in any parameters. Although sensitivity analysis of the net reproductive rate *R*_0_ has been presented before (Matser et al. 2009; Caswell 2009), there has been no such analysis for the variance or other measures of variation in LRO.

We derive sensitivity formulae using matrix calculus formalism (Caswell 2007, 2008, 2009, 2012; Caswell and Salguero-Gómez 2013). Let ***𝜃*** be a vector (*p* × 1) of parameters of interest; these could be mortalities, transition probabilities, means or variances of fertility, etc. The sensitivity of the moment vector $\tilde {\boldsymbol {\rho }}_{m}$ to the parameter vector ***𝜃*** is the derivative matrix

26 $$ {d \tilde{\boldsymbol{\rho}}_{m} \over d \boldsymbol{\theta}^{\textsf{T}}} = \left( \begin{array}{c} {d \tilde{\boldsymbol{\rho}}_{m}(i) \over d \boldsymbol{\theta}(j)} \end{array}\right) \qquad \tau \times p . $$

That is, the (*i*,*j*) entry of this sensitivity matrix is the derivative of the *i*th entry of $\tilde {\boldsymbol {\rho }}_{m}$ with respect to the *j*th entry of ***𝜃***.

To write the sensitivity of the $\tilde {\boldsymbol {\rho }}_{m}$, let us define the following matrices.

27 $$\begin{array}{@{}rcl@{}} \textbf{V}_{i} &=& \left( \mathbf{1}_{s}^{\textsf{T}} \otimes \textbf{Z} \right) \textbf{K}_{ss} \left[ \rule{0in}{3ex} \mathcal{D} \left( \text{vec}\ \textbf{R}_{i} \right) {d \text{vec}\ \textbf{P} \over d \boldsymbol{\theta}^{\textsf{T}}} \,+\, \mathcal{D} (\text{vec}\ \textbf{P}) {d \text{vec}\ \textbf{R}_{i} \over d \boldsymbol{\theta}} \right] \end{array} $$

28 $$\begin{array}{@{}rcl@{}}\textbf{W}_{i,j} &=& \left( \tilde{\boldsymbol{\rho}}_{i}^{\textsf{T}} \otimes \textbf{I}_{\tau} \right) \textbf{K}_{\tau \tau} \left[\mathcal{D} \left( \text{vec}\ \tilde{\textbf{R}}_{j} \right) {d \text{vec}\ \textbf{U} \over d \boldsymbol{\theta}^{\textsf{T}}} \,+\, \mathcal{D} \left( \text{vec}\ \textbf{U} \right) {d \text{vec}\ \tilde{\textbf{R}}_{j} \over d \boldsymbol{\theta}^{\textsf{T}}} \right]\\&& + \left( \textbf{U} \circ \tilde{\textbf{R}}_{j} \right)^{\textsf{T}} {d \tilde{\boldsymbol{\rho}}_{i} \over d \boldsymbol{\theta}^{\textsf{T}}} \end{array} $$

29 $$\begin{array}{@{}rcl@{}} \textbf{X}_{i} &=& \left( \tilde{\boldsymbol{\rho}}_{i}^{\textsf{T}} \otimes \textbf{I}_{\tau} \right) \textbf{K}_{\tau \tau} {d \text{vec} \ \textbf{U} \over d \boldsymbol{\theta}^{\textsf{T}}}. \end{array} $$

In Eqs. 27–29, **K**_*mn*_ is the vec-permutation matrix of order (*m*,*n*); see Henderson and Searle (1981) and Magnus and Neudecker (1979).

### **Theorem 2**

Let **P**, **U**, and **M** *define the absorbing Markov chain in Eq*. 3, with *τ* transient states, *α* *absorbing states, and s* = *τ* + *α* total states. Let **R**_*i*_ *contain the ith* *moments of the reproductive rewards corresponding to each transition. Let* ***𝜃*** *be a vector of parameters. The vector* $\tilde {\boldsymbol {\rho }}_{m}$ *contains the m* *th moments of remaining LRO for each of the τ transient stages. The sensitivity of* $\tilde {\boldsymbol {\rho }}_{m}$ *to* ***𝜃*** *is*

30 $$ {d \tilde{\boldsymbol{\rho}}_{m} \over d \boldsymbol{\theta}^{\text{\textsf{T}}}} = \text{\textbf{N}}^{\text{\textsf{T}}} \left[\text{\textbf{V}}_{m} + \sum\limits_{k=1}^{m-1} {m \choose k} \text{\textbf{W}}_{k,m-k} + \text{\textbf{X}}_{m} \right] \qquad m=1,2,\ldots  $$

### *Proof*

Proof is given in Appendix A. □

The sensitivities of the first three moments of LRO are of particular interest; they are

31 $$\begin{array}{@{}rcl@{}} {d \tilde{\boldsymbol{\rho}}_{1} \over d \boldsymbol{\theta}^{\textsf{T}}} &=& \textbf{N}^{\textsf{T}} \left( \textbf{V}_{1} + \textbf{X}_{1} \right) \end{array} $$

32 $$\begin{array}{@{}rcl@{}} {d \tilde{\boldsymbol{\rho}}_{2} \over d \boldsymbol{\theta}^{\textsf{T}}} &=& \textbf{N}^{\textsf{T}} \left( \textbf{V}_{2} + 2 \textbf{W}_{1,1} + \textbf{X}_{2} \right) \end{array} $$

33 $$\begin{array}{@{}rcl@{}} {d \tilde{\boldsymbol{\rho}}_{3} \over d \boldsymbol{\theta}^{\textsf{T}}} &=& \textbf{N}^{\textsf{T}} \left( \textbf{V}_{3} + 3 \textbf{W}_{1,2} + 3 \textbf{W}_{2,1} + \textbf{X}_{3} \right). \end{array} $$

If, as is often the case, the model includes only a single absorbing state (death), then **M** is a (1 × *τ*) matrix, given by

34 $$ \textbf{M} = \mathbf{1}_{\tau}^{\textsf{T}} - \mathbf{1}_{\tau}^{\textsf{T}} \textbf{U} \qquad 1 \times \tau .  $$

In this case, the derivative of **P** that appears in Eq. 27 is

35 $$ {d \text{vec}\ \textbf{P} \over d \boldsymbol{\theta}^{\textsf{T}}} = \left[\textbf{C}_{1} - \textbf{C}_{2} \left( \textbf{I}_{\tau} \otimes \mathbf{1}_{\tau}^{\textsf{T}} \right) \right] {d \text{vec}\ \textbf{U} \over d \boldsymbol{\theta}^{\textsf{T}}}  $$

where

36 $$\begin{array}{@{}rcl@{}} \textbf{C}_{1} &=&\left( \begin{array}{l} \textbf{I}_{\tau \times \tau} \\ \textbf{0}_{1 \times \tau} \end{array}\right) \otimes \left( \begin{array}{l} \textbf{I}_{\tau \times \tau} \\ \textbf{0}_{1 \times \tau} \end{array}\right) \end{array} $$

37 $$\begin{array}{@{}rcl@{}} \textbf{C}_{2} &=&\left( \begin{array}{l} \textbf{I}_{\tau \times \tau} \\ \textbf{0}_{1 \times \tau} \end{array}\right) \otimes \left( \begin{array}{l} \textbf{0}_{\tau \times 1} \\ \textbf{I}_{1 \times 1} \end{array}\right) \end{array} $$

Caswell and Van Daalen (2016). This permits expressing all the effects of ***𝜃*** on the transition matrix **P** in terms of effects on **U** (see Appendix A).

### Sensitivity of the statistics of LRO

The moments $\tilde {\boldsymbol {\rho }}_{i}$ provide the statistics (21)–(24) describing the inter-individual variability of LRO, including the variance, standard deviation, coefficient of variation, and the scaled variance (Crow’s index) (Caswell 2011; Van Daalen and Caswell 2015).

The sensitivities of these quantities are

38 $$\begin{array}{@{}rcl@{}} {d V(\tilde{\boldsymbol{\rho}}) \over d \boldsymbol{\theta}^{\textsf{T}}} &=& {d \tilde{\boldsymbol{\rho}}_{2} \over d \boldsymbol{\theta}^{\textsf{T}}} - 2 \mathcal{D}(\tilde{\boldsymbol{\rho}}_{1}) \; {d \tilde{\boldsymbol{\rho}}_{1} \over d \boldsymbol{\theta}^{\textsf{T}}} \end{array} $$

39 $$\begin{array}{@{}rcl@{}} {d SD(\tilde{\boldsymbol{\rho}}) \over d \boldsymbol{\theta}^{\textsf{T}}} &=& \frac{1}{2} \mathcal{D} \left[ \rule{0in}{2ex} SD(\tilde{\boldsymbol{\rho}}) \right]^{-1} \; {d V(\tilde{\boldsymbol{\rho}}) \over d \boldsymbol{\theta}^{\textsf{T}}} \end{array} $$

40 $$\begin{array}{@{}rcl@{}} {d CV(\tilde{\boldsymbol{\rho}}) \over d \boldsymbol{\theta}^{\textsf{T}}} &=& \mathcal{D} \left( \tilde{\boldsymbol{\rho}}_{1} \right)^{-1} \; {d SD(\tilde{\boldsymbol{\rho}}) \over d \boldsymbol{\theta}^{\textsf{T}}}\\&& \,-\, \left( \rule{0in}{2.5ex}SD (\tilde{\boldsymbol{\rho}}) \otimes \textbf{I}_{\tau} \right) \mathcal{D} \left( \tilde{\boldsymbol{\rho}}_{1} \right)^{-2} \; {d \tilde{\boldsymbol{\rho}}_{1} \over d \boldsymbol{\theta}^{\textsf{T}}} \end{array} $$

41 $$\begin{array}{@{}rcl@{}} {d \mathcal{I} \over d \boldsymbol{\theta}^{\textsf{T}}} &=& 2 \mathcal{D} \left[ \rule{0in}{2ex}CV(\tilde{\boldsymbol{\rho}}) \right] \; {d CV(\tilde{\boldsymbol{\rho}}) \over d \boldsymbol{\theta}^{\textsf{T}}} . \end{array} $$

For derivations, see Appendix A.

### Elasticity

The derivatives in Theorem 2 and Eqs. 38–41 measure the effect of an additive perturbation of the parameter vector ***𝜃***. Elasticities, which measure the proportional change resulting from a proportional change in ***𝜃***, are easily calculated. Let *ξ* be any quantity calculated from the demographic model. The elasticity of *ξ* with respect to ***𝜃*** is the matrix

42 $$ {\varepsilon \xi \over \varepsilon \boldsymbol{\theta}^{\textsf{T}}} = \mathcal{D}( \xi)^{-1} \; {d \xi \over d \boldsymbol{\theta}^{\textsf{T}}} \; \mathcal{D}(\boldsymbol{\theta}) .  $$

As usual, elasticity calculations can be applied only if *ξ* > 0 and ***𝜃*** ≥ 0.

### Some special perturbations

Here we consider some perturbations that are of special interest: the survival and transitions in stage-classified models and sensitivity to means and variances of stage-specific fertility.

#### Mortality and transitions in stage-classified models

In an age-classified model, the transient matrix **U** is completely determined by the mortality schedule (see “Age-classified human populations” for age-classified examples). In a stage-classified model, however, **U** depends on both survival and probabilities of transitions among stages. To calculate sensitivities of LRO to survival and transition probabilities, we write

43 $$ \textbf{U} = \textbf{G} {\boldsymbol{\Sigma}}  $$

where ***Σ*** has the survival probabilities ***σ*** on the diagonal; i.e., $\boldsymbol {\Sigma } = \mathcal {D}(\boldsymbol {\sigma })$, and **G** is a matrix of transition probabilities conditional on survival. Differentiating (43) gives

44 $$ d \text{vec}\ \textbf{U} = \left( \textbf{I}_{\tau} \otimes \textbf{G} \right) d \text{vec}\ \boldsymbol{\Sigma} + \left( \boldsymbol{\Sigma} \otimes \textbf{I}_{\tau} \right) d \text{vec}\ \textbf{G}.  $$

Writing $\mathcal {D} (d \boldsymbol \sigma ) = \textbf {I} \circ \left (\mathbf {1}_{\tau } d \boldsymbol \sigma ^{\textsf {T}} \right )$ and simplifying lead to

45 $$ {d \text{vec}\ \textbf{U} \over d \boldsymbol\sigma^{\textsf{T}}} = \left( \textbf{I}_{\tau} \otimes \textbf{G} \right) \mathcal{D}(\text{vec}\ \textbf{I}) \left( \textbf{I}_{\tau} \otimes \mathbf{1}_{\tau} \right), $$

and since ***σ*** = exp(−***μ***),

46 $$ {d \text{vec}\ \textbf{U} \over d \boldsymbol\mu^{\textsf{T}}} = - {d \text{vec}\ \textbf{U} \over d \boldsymbol\sigma^{\textsf{T}}} \mathcal{D}(\boldsymbol\sigma) .  $$

From (44), the sensitivity of **U** to the growth matrix **G** is

47 $$ \frac{d \text{vec}\ \textbf{U}}{d \text{vec}^{\textsf{T}} \textbf{G}} = ( \boldsymbol{\Sigma}^{\textsf{T}} \otimes \textbf{I}). $$

However, **G** is column-stochastic; i.e., its columns must sum to 1. An arbitrary perturbation of **G** would result in the loss of the column-stochastic property. The only relevant perturbations are those that maintain the column sums. Thus, perturbations of the entry *g*_*ij*_ must be compensated for by changes in other elements of column *j*. Caswell (2001, 2013) presents a method for compensating for perturbation of each entry in a column-stochastic matrix in a way that maintains the proportional structure of the column. In matrix notation, we write

48 $$ \left. \frac{d \text{vec}\ \textbf{U}} {d \text{vec}^{\textsf{T}}\textbf{G}} \right|_{\text{comp}} = \frac{d \text{vec}\ \textbf{U}} {d \text{vec}^{\textsf{T}} \textbf{G}} \; {d\text{vec}\ \textbf{G} \over d \text{vec}^{\textsf{T}} \boldsymbol{\Theta}} $$

where the matrix ***Θ*** is a matrix of perturbations that include the compensation for the column sums. The matrix of derivatives of **G** with respect to ***Θ*** is given by Caswell (2013). Let **g**_*i*_ denote column *i* of **G**; then

49 $$ {d \text{vec}\ \textbf{G} \over d \text{vec}^{\textsf{T}} \boldsymbol{\Theta}} = \textbf{I}_{\tau^2} - \sum\limits_{i=1}^{\tau} \textbf{E}_{ii} \otimes \left[ \mathcal{D} \left( \textbf g_{i}\right) \; \textbf{C} \; \mathcal{D} \left( \mathbf{1} - \textbf g_{i} \right)^{-1}\right]  $$

where **C** = **E** −**I**.

#### Sensitivity to means and variances of fertility

Perturbations of fertility appear in Eqs. 27–29 as derivatives of the reproductive reward matrices **R**_*i*_. When the distributions of stage-specific fertilities are specified by a parametric distribution, the moments may be linked, so that changes in mean fertility also affect the variance (e.g., in the Poisson distribution, the variance is equal to the mean). Sometimes, however, it is of interest to treat the mean and variance of fertility as independent traits and calculate the sensitivity of LRO to the mean, holding the variance fixed, and to the variance, holding the mean fixed. This subtle but important distinction was emphasized by Tuljapurkar et al. (2003) and Haridas and Tuljapurkar (2005) in the context of the elasticity of the stochastic growth rate to the entries of a stochastically varying matrix. One might compute the effect of changing one of the matrix entries (perhaps by a change in energy allocation strategy), recognizing that this would change both the mean and the variance. Or, one might be interested in the effects of variance per se and manipulate the moments to calculate elasticities with respect to the mean and variance independently.

As in Eq. 11, suppose that fertility is defined by a fertility vector **f** with first and second moments **f**_1_ and **f**_2_, and a variance vector

50 $$ \textbf{v} = \textbf{f}_{2} - \left( \textbf{f}_{1} \circ \textbf{f}_{1} \right).  $$

The first and second moment matrices are given by

51 $$ \textbf{R}_{i} = \mathbf{1}_{s} \textbf{f}_{i}^{\textsf{T}} \textbf{Z} \qquad i=1,2. $$

##### Sensitivity to mean fertility, variance fixed

From Eq. 50, it follows that

52 $$ d \textbf{v} = d \textbf{f}_{2} - 2 \mathcal{D}(\textbf{f}_{1}) d \textbf{f}_{1} .  $$

To hold the variance fixed, we require *d* **v** = 0, which implies that

53 $$ {d \textbf{f}_{2} \over d \textbf{f}_{1}^{\textsf{T}}}=2 \mathcal{D}(\textbf{f}_{1}).  $$

To evaluate sensitivity to the mean, we set the parameter vector ***𝜃*** = **f**_1_, subject to Eq. 53, and obtain

54 $$\begin{array}{@{}rcl@{}} \left. {d \text{vec}\ \textbf{R}_{1} \over d \boldsymbol{\theta}^{\textsf{T}}} \right|_{d \textbf{v} =0} &=& \left( \textbf{Z}^{\textsf{T}} \otimes \textbf{1}_{s}\right) \end{array} $$

55 $$\begin{array}{@{}rcl@{}} \left. {d \text{vec}\ \textbf{R}_{2} \over d \boldsymbol{\theta}^{\textsf{T}}} \right|_{d\textbf{v}=0} &=& \left( \textbf{Z}^{\textsf{T}} \otimes \textbf{1}_{s} \right) {d \textbf{f}_{2} \over d \textbf{f}_{1}} \end{array} $$

56 $$\begin{array}{@{}rcl@{}} &=&2 \left( \textbf{Z}^{\textsf{T}} \otimes \textbf{1}_{s} \right) \mathcal{D}(\textbf{f}_{1}) . \end{array} $$

Substituting these expressions into Eqs. 31 and 32, and then into Eq. 38, we obtain the sensitivity of the mean and variance of LRO to changes in mean fertility, with variance in fertility held constant.

##### Sensitivity to variance in fertility, mean fixed

To hold the mean fixed, we require that *d* **f**_1_ = 0, in which case (52) implies that

57 $$ {d \textbf{f}_{2} \over d \textbf{v}^{\textsf{T}}} = \textbf{I}_{\tau}. $$

Now we set the parameter vector ***𝜃*** = **v** to obtain

58 $$\begin{array}{@{}rcl@{}} \left. {d \text{vec}\ \textbf{R}_{1} \over d \boldsymbol{\theta}^{\textsf{T}}} \right|_{d \textbf{f}_{1}=0} &=& \boldsymbol{0}_{s \times \tau} \end{array} $$

59 $$\begin{array}{@{}rcl@{}} \left. {d \text{vec}\ \textbf{R}_{2} \over d \boldsymbol{\theta}^{\textsf{T}}} \right|_{d \textbf{f}_{1}=0} &=& \left( \textbf{Z}^{\textsf{T}} \otimes \textbf{1}_{s} \right) {d \textbf{f}_{2} \over d \textbf{v}^{\textsf{T}}} \end{array} $$

60 $$\begin{array}{@{}rcl@{}} &=&\left( \textbf{Z}^{\textsf{T}} \otimes \textbf{1}_{s} \right) . \end{array} $$

Substituting these expressions into Eqs. 31 and 32, and then into Eq. 38, gives the sensitivity of the mean and variance of LRO to changes in the variance in fertility, with mean fertility held constant.

## A protocol for the analysis of lifetime reproductive output

The results presented to this point provide a protocol for analysis of lifetime reproductive output, applicable to any matrix population model. A stepwise version of this protocol is given in Table 1. In the next section, we present age-classified and stage-classified examples of the analysis.

**Table 1:** A step-by-step protocol for analysis of lifetime reproductive output and its sensitivity, from any stage- or age-classified matrix population model

1. Obtain a transition matrix **U**, perhaps from decomposing a population projection matrix as **A** = **U** + **F**.
2. Locate reproductive transitions.
(a) If fertility is transition specific, identify the transitions (e.g., to reproductive states).
(b) If fertility is stage-specific, extract the vector **f** from **F**.
3. Obtain statistical moments of fertility:
(a) From empirical measurements of the moments of stage-specific fertility, or
(b) From an assumption of Bernoulli [see equation (5)], or Poisson [ see Eqs. 6 and 7], or fixed [see Eqs. 8 and 9] reproduction.
4. Construct reward matrices from Eq. 11.
5. Compute desired moments of LRO from Eqs. 16–19.
6. Compute desired statistics of LRO from Eqs. 21–24.
7. Sensitivity analysis
(a) Specify parameter vector ***𝜃*** of interest
(b) Calculate derivatives of **U**, and **R**_*i*_ to ***𝜃***. Take advantage of Eqs. 35 or 90–92 to compute derivatives of **P** to ***𝜃***.
(c) If the matrix is stage-classified,
i. Decompose **U** = **G**Σ.
ii. Use Eq. 46 to compute the derivative of **U** to mortality.
iii. Use Eq. 49 to compute the derivative of **G** to ***𝜃***, including compensation to preserve column sums of **G**
(d) Compute derivatives of the moment vectors $\tilde {\boldsymbol {\rho }}_{i}$ for the moments of interest (*i* = 1, 2 suffice to analyze the variance, standard deviation,
CV, and $\mathcal {I}$).
i. Compute **V**, **W**, and **X** using Eqs. 27–29.
ii. Compute derivatives of $\tilde {\boldsymbol {\rho }}_{i}$ using Theorem 2.
(e) Compute sensitivity of desired statistics of LRO using Eqs. 38–41.
(f) If desired, compute elasticities of statistics of LRO using Eq. 42.

## Examples

This section presents examples of the calculation of the statistical properties of lifetime reproductive output and its subsequent sensitivity analysis, for both age-classified and stage-classified population. In “Age-classified human populations,” we analyze a set of age-classified human populations that span a wide range of demographic conditions. In “A stage-classified tree population,” we analyze a size-classified model for Canadian hemlock (*Tsuga canadensis*), a coniferous tree.

### Age-classified human populations

The transition matrix **U** for an age-classified model contains survival probabilities on the subdiagonal and zeros elsewhere. One absorbing state, death, is included, and the mortality matrix **M** is calculated according to Eq. 34.

We ignore multiple births and treat the entries of the fertility vector **f** as the probability of producing a single offspring. The offspring production is given by the reward *r*_*ij*_, following a Bernoulli distribution,

61 $$ r_{ij} = \left\{ \begin{array}{l} 1 \text{ with probability} \ f_{j} \\ 0 \text{ with probability}\ (1-f_{j}) \end{array} \right. . $$

The matrix **R**_1_ containing the first moment of the reward matrix is built from the fertility vector using Eq. 11. The higher moments of the reward matrix follow from the Bernoulli model of reproduction, in which the higher moments are all equal to the first, as in Eq. 5, so that **R**_1_ = **R**_2_ = **R**_3_.

We present results for nine populations: the Netherlands (1950 and 2009), Sweden (1891 and 2010), Japan (1947 and 2009), two hunter-gather populations (the Ache of subtropical Paraguay (Gurven and Kaplan 2007; Hill and Hurtado 1996) and the Hadza of the Tanzanian savanna Blurton Jones 2011), and the Hutterites of North America. The Netherlands, Sweden, and Japan are typical of developed countries progressing through the demographic transition. The hunter-gatherer populations have higher mortality, lower life expectancy, and higher fertility than the developed countries. The Hutterites, an Anabaptist religious community in the United States and Canada, are known for having the highest total fertility for any known human population (Eaton and Mayer 1953), but are assumed to have experienced mortality rates similar to that of the USA around 1946–1950.

Data for the Netherlands, Sweden, and Japan were obtained from the Human Mortality Database (2013) and the Human Fertility Database (2013). Data for the Ache were obtained from Gurven and Kaplan (2007) and Hill and Hurtado (1996), and for the Hadza from Blurton Jones (2011). The fertility and mortality schedules for the Hutterites were taken from Eaton and Mayer (1953).

#### Variance in LRO

Using Theorem 1 and Eqs. 21–24, and following the protocol in Table 1, we computed the mean, variance (both within and between pathways), standard deviation, coefficient of variation, and Crow’s $\mathcal {I}$ from **U**, **R**_1_, and **R**_2_. We also calculated life expectancy as the column sums of the fundamental matrix. The results are given in Table 2.

**Table 2:** The statistics of lifetime reproductive output for the Netherlands (NLD), Sweden (SWE), and Japan (JPN), with two points in time for each country, two hunter-gatherer populations, the Hadza and the Ache, and a population of high-fertility Hutterites

Population	Mean	*V*	*V*_between_ (*%*)	*V*_within_ (*%*)	SD	CV	$\mathcal {I}$	Life exp.
NLD 1950	2.96	2.91	12.5	87.5	1.71	0.58	0.33	73.1
NLD 2009	1.78	1.61	1.4	98.6	1.27	0.72	0.51	83.1
SWE 1891	3.00	5.60	55.6	44.4	2.37	0.79	0.62	53.0
SWE 2010	1.97	1.79	1.4	98.6	1.34	0.68	0.46	84.0
JPN 1947	3.50	6.10	54.8	45.2	2.47	0.71	0.50	54.2
JPN 2009	1.35	1.26	0.9	99.1	1.12	0.83	0.69	86.9
Hadza	3.13	11.30	78.9	21.1	3.36	1.07	1.15	34.6
Ache	4.48	17.23	81.1	18.9	4.15	0.93	0.86	38.0
Hutterites	7.53	8.58	41.3	58.7	2.93	0.39	0.15	70.0

Not surprisingly, recent populations in developed countries have higher longevity and lower mean lifetime reproductive output. The hunter-gatherer and Hutterite populations show the highest mean LRO. In each of the developed countries (the Netherlands, Sweden, and Japan), the reductions in mean LRO and increases in life expectancy are accompanied by reductions in the variance in LRO. The Ache and Hadza show the highest variance in LRO, and the variance for the Hutterites is higher than any of the developed countries.

Most of the variance in the hunter-gatherer populations is due to variance among pathways, which in an age-classified model is determined by survival from birth through the reproductive ages. The most recent years in developed countries show extremely low between-pathway variance because almost all women survive through their reproductive years (Van Daalen and Caswell 2015). The Ache and Hadza, with the lowest life expectancy, show very high *V*_between_. The Hutterites have a long life expectancy, but their high fertility amplifies the effect of differences in longevity, so *V*_between_ is similar to that of Japan in 1947 and Sweden in 1891.

The opportunity for selection $\mathcal {I}$ varies less (about sevenfold) among these populations than does the variance in LRO (about 13-fold). The Hutterites showed the lowest opportunity for selection of all the populations we included, yet the other high-fertility populations, the Ache and the Hadza, show the highest values for the opportunity for selection. Taking into account the fact that the hunter-gatherer populations have the highest variances in LRO, mostly due to variation in the pathways individuals take through life, we posit that the high opportunity for selection reflects room for improvement in survival rates from birth to reproductive ages.

The variance in LRO documented in Table 2 is calculated on the assumption that every individual experiences the same vital rates at every age and is thus due to individual stochasticity. Crow’s $\mathcal {I}$ is a measure of the potential relative increase in fitness per generation, but the variance here is stochastic, not genetic, so the opportunity for selection is only apparent, not real.

#### Sensitivity analysis

The age-classified models for human populations are parameterized by the mortality rate vector ***μ*** and the mean fertility vector **f**. The sensitivity of the statistics of LRO to these parameters, obtained from Theorem 2 and Eqs. 38–41, requires *d*vec **U**/*d* ***μ*** ^T^ and *d*vec **R**_*i*_/*d* **f** ^T^ for *i* = 1,2. The derivative of **U** is

62 $$ {d \text{vec}\ \textbf{U} \over d \boldsymbol\mu^{\textsf{T}}} = - \mathcal{D}(\text{vec}\ \textbf Y)(\textbf{I} \otimes \mathbf{1})\mathcal{D}(\textbf{P}) $$

where **Y** is an indicator matrix defining the subdiagonal structure of **U**, and **P** = exp(−***μ***) is the vector of survival probabilities. The Bernoulli distribution assumption implies that the derivatives of the first and second (and all other) moments are equal, with

63 $$ {d \textbf{R}_{1} \over d \textbf{f}^{\textsf{T}}} = \left( \textbf{Z}^{\textsf{T}} \otimes \textbf{1}_{s} \right)  $$

(for derivations, see Appendix A).

**Fig. 1:**
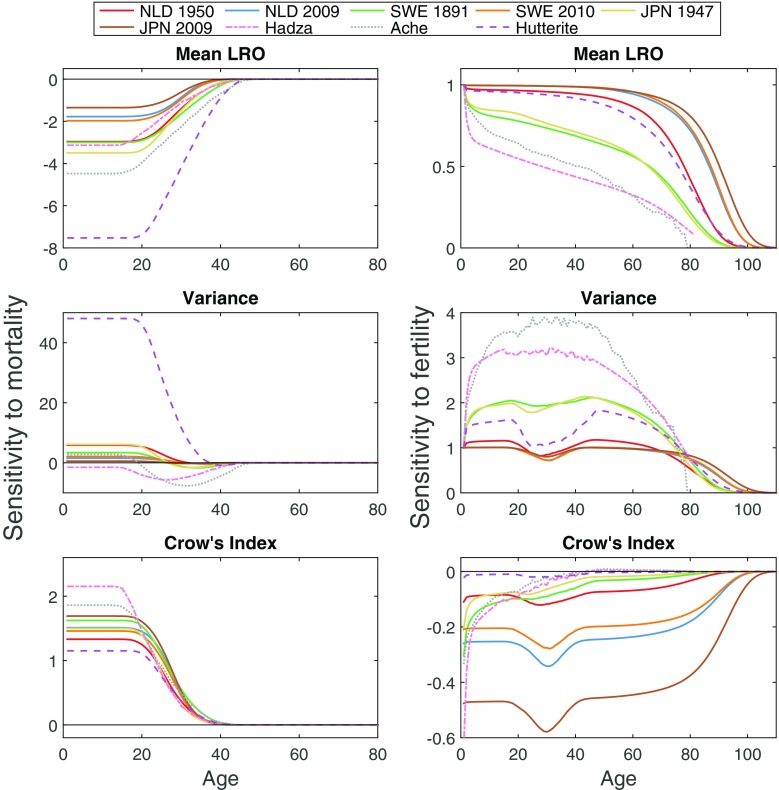
Sensitivity of mean LRO, variance in LRO, and Crow’s index to changes in age-specific mortality (*left column*) and age-specific fertility (*right column*) for nine human populations

The sensitivity of mean LRO, variance in LRO, and Crow’s $\mathcal {I}$ to mortality and fertility are shown in Fig. 1. Not surprisingly, increased mortality at any age up to the end of the reproductive period reduces mean LRO, while increased fertility increases mean LRO. The mortality effect is greatest for the Hutterites, because their high fertility magnifies the impact of mortality changes, and least for the recent developed countries. The sensitivity of mean LRO to fertility is given by the survivorship function and thus is smallest for the Ache and Hadza. It is highest for recent developed countries in which low mortality means that almost everyone would survive to benefit from an increase in fertility.

The variance in LRO increases with an increase in mortality rate for most populations; the effect is greatest for the Hutterites (Fig. 1). For the developed countries, the sensitivity is positive over most of the reproductive ages. For the Hadza and the Ache, variance in LRO decreases with higher age-specific mortality rates. Variance in LRO increases with increasing fertility rates for all countries. The Hutterites, Sweden, the Netherlands, and Japan show a reduced sensitivity of variance in LRO to fertility around the reproductive ages.

Crow’s opportunity for selection $\mathcal {I}$ combines both the mean and the variance. Increased mortality during the reproductive period increases $\mathcal {I}$ in all the populations. It is most sensitive to mortality in the Ache and Hadza populations and least sensitive in the Hutterites. An increase in fertility reduces $\mathcal {I}$ in all the populations. Thus, the net result of environmental changes that affect both mortality and fertility cannot be predicted a priori.

**Fig. 2:**
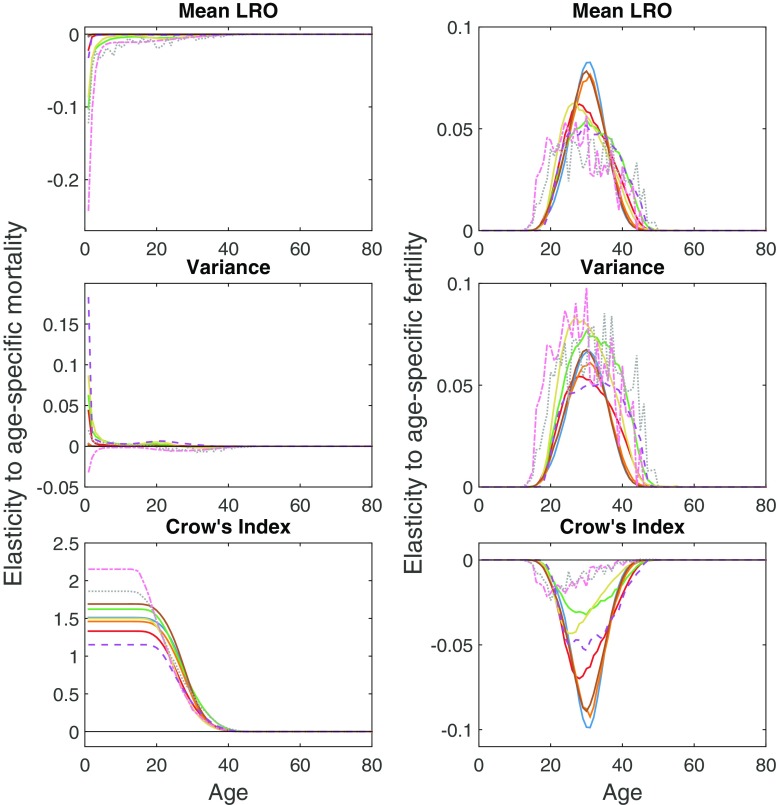
Elasticity of mean LRO, variance in LRO, and Crow’s index to changes in age-specific mortality (*left column*) and age-specific fertility (*right column*) for nine human populations

Both mortality and fertility vary widely across ages in these populations, so it may be useful to standardize the responses by calculating elasticities (Fig. 2). The elasticities of the mean and variance of LRO with respect to mortality are generally low, except for effects of infant mortality, especially for the hunter-gatherer populations, in which infant mortality is high. However, the elasticity of Crow’s $\mathcal {I}$ is large and positive, in fact, the largest of any of the elasticities obtained. The elasticities with respect to fertility are naturally confined to the reproductive ages. Proportional increases in fertility increase the mean and variance of LRO, but reduce the value of Crow’s $\mathcal {I}$.

### A stage-classified tree population

As an example of a typical stage-classified population, we analyze a model for the Canadian hemlock (*T. canadensis* L.). Lamar and McGraw (2005) developed a model based on six size classes (from < 5.0 cm dbh (diameter at breast height) to > 42.5 cm dbh). They reported population projection matrices for trees in a low-disturbance plot in Shenandoah National Park in the eastern USA, between 1997 and 1999. We obtained **U** and **F** from the mean population matrix **A** obtained from the Compadre Plant Matrix Database (Compadre Plant Matrix Database 2014).

64 $$ \textbf{A} = \left( \begin{array}{cccccc} 0.90 & 0 & 0.30 & 0.77 & 1.96 & 6.03 \\ 0.004 & 0.96 & 0 & 0 & 0 & 0 \\ 0 & 0.012 & 0.97 & 0 & 0 & 0 \\ 0 & 0 & 0.017 & 0.98 & 0 & 0 \\ 0 & 0 & 0 & 0.012 & 0.96 & 0 \\ 0 & 0 & 0 & 0 & 0.018 & 0.99 \end{array}\right) $$

Reproduction was measured as new recruits (rather than seeds or seedlings) per individual, per year, as a function of size (Lamar and McGraw 2005). In the absence of information on the empirical distribution of size-specific fertility, we use the Poisson distribution to define the reward moment matrices **R**_1_ and **R**_2_ following Eqs. 11 and 6.

#### Results

The mean, variance, and other statistics of lifetime reproductive output for *T. canadensis*, obtained using Theorem 1 and Eqs. 21–24, are shown in Table 3. Because of the high mortality of small trees, the mean LRO is small and life expectancy is only 12 years. However, the variance in LRO is very high, as is the apparent opportunity for selection $\mathcal {I}$. The variance in LRO exceeds that for any of the human populations by two orders of magnitude. The value of $\mathcal {I}$ is 600 times higher than the highest for human populations in Table 2. Almost all of the variance in LRO is due to variance among pathways. The variance due to stochastic rewards along those pathways is small and approximately equal to the mean, which reflects the assumption of Poisson distributed fertility. It is swamped by the huge differences among pathways of trees that die small and those that become large and reproductive.

**Table 3:** The statistics of lifetime reproductive output for *T. canadensis*

Mean	*V*	*V*_between_ (*%*)	*V*_within_ (*%*)	SD	CV	$\mathcal {I}$	Life exp.
1.42	1.41 × 10^3^	99.9	0.1	37.54	26.39	696.23	12.16

#### Sensitivity analysis

As in Eq. 43, the transition matrix **U** is the product of the survival matrix ***Σ*** and a growth matrix **G**. The derivations of the equations below are presented in Appendix A. The derivative of the transition matrix **U** with respect to mortality is

65 $$\begin{array}{@{}rcl@{}} \frac{d \text{vec} \ \textbf{U}}{d \boldsymbol{\mu}^{\textsf{T}}} = \frac{d \text{vec} \ \textbf{U}}{d\text{vec}^{\textsf{T}} \boldsymbol{\Sigma}} \frac{d\text{vec} \boldsymbol{\Sigma}}{d \boldsymbol{\mu}^{\textsf{T}}} = - (\textbf{I} \otimes \textbf{G})\mathcal{D}(\text{vec} \ \textbf{I}) (\textbf{I} \otimes \mathbf{1})\mathcal{D}(\textbf{p}). \end{array} $$

The sensitivity of **U** to the growth matrix **G**, maintaining the column sums, is

66 $$ \left. \frac{d \text{vec} \ \textbf{U}} {d \text{vec}^{\textsf{T}} \textbf{G}} \right|_{\text{comp}} = \left( \boldsymbol{\Sigma} \otimes \textbf{I} \right) \frac{d \text{vec} \ \textbf{G}} {d \text{vec}^{\textsf{T}} \boldsymbol{\Theta}}. $$

where *d*vec **G**/*d*vec^T^ ***Θ*** is given by Eq. 49.

The sensitivity of LRO to fertility is determined by the derivatives of the **R**_*i*_ to fertility. The derivative of the matrix **R**_1_ of mean fertility is given by Eq. 63. The derivatives of **R**_2_ and **R**_3_ with respect to **f** are calculated from the Poisson distribution using Eqs. 6 and 7:

67 $$\begin{array}{@{}rcl@{}} \frac{d \text{vec} \ \textbf{R}_{2}}{d \textbf{f}^{\textsf{T}}} &=& \left[\textbf{I}_{s^{2}}+ 2 \mathcal{D}(\text{vec}\ \textbf{R}_{1})\right] \left( \left.\textbf{Z}^{\textsf{T}} \otimes \mathbf{1}_{s} \right)\right) \end{array} $$

68 $$\begin{array}{@{}rcl@{}} \frac{d \text{vec} \ \textbf{R}_{3}}{d \textbf{f}^{\textsf{T}}} &=&\left[\textbf{I}_{s^{2}}\,+\, 6 \mathcal{D}(\text{vec} \ \textbf{R}_{1}) \,+\, 3 \mathcal{D} \left( \text{vec}\ (\textbf{R}_{1} \circ \textbf{R}_{1}) \right) \right] \left( \textbf{Z}^{\textsf{T}} \otimes \mathbf{1}_{s} \right) . \end{array} $$

Incorporating (67) and (68) into Eqs. 27–29 and applying Theorem 2 provides the sensitivity of lifetime reproductive output to mortality, fertility, and the growth matrix (see Appendix A for the derivations). To make comparisons across these variables measured on different scales, we calculate the elasticity or proportional sensitivity of LRO using Eq. 42.

**Fig. 3:**
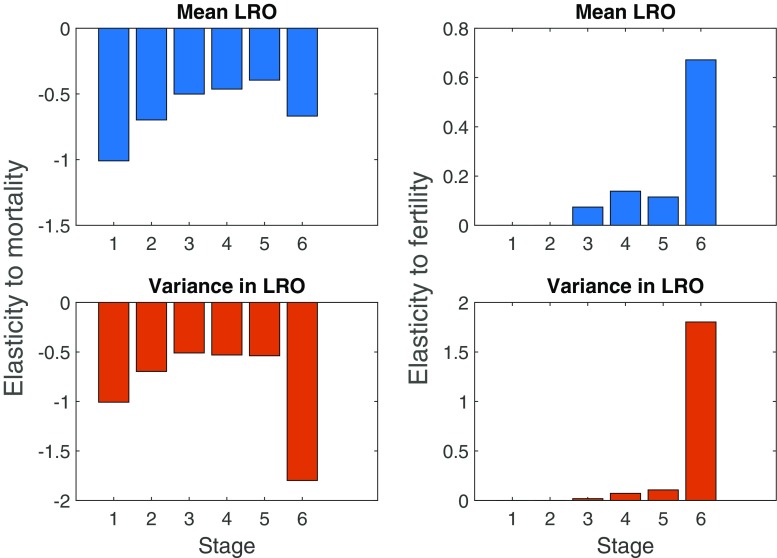
Elasticity of mean LRO and variance in LRO to changes in stage-specific mortality (*left column*) and stage-specific fertility (*right column*) for *T. canadensis*

An increase in mortality in the first size-class or the last size-class reduces both mean lifetime reproduction of Hemlock individuals and variance in LRO (Fig. 3). Increasing fertility rates increases both mean and variance in LRO for trees in the last size-class. These are the trees with the highest survival and fertility rates.

**Fig. 4:**
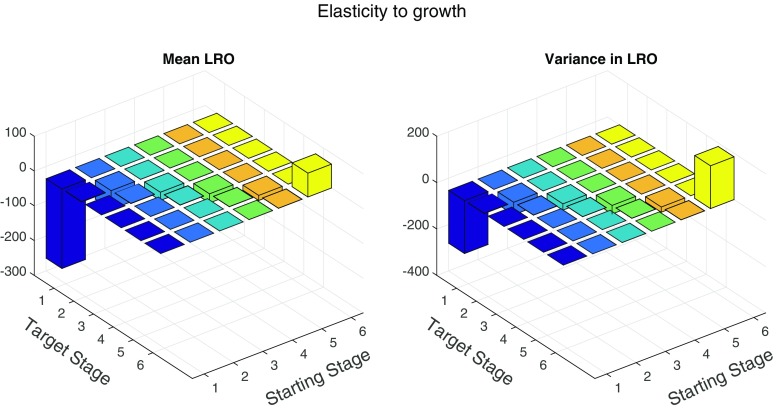
Sensitivity of the mean LRO and variance in LRO of *Tsuga canadensis* to the growth matrix (*left* and *right* panels, respectively)

The elasticity of the mean and variance to the growth matrix **G** is shown in Fig. 4. The results are dominated by the extremely large negative elasticities to the probability of remaining in stage 1 (thereby reducing growth) and the large positive elasticity to the probability of remaining in the largest size class. Increasing the probability of staying in any size-class (again, reducing growth) reduces both mean and variance in LRO.

The mean and variance, the contribution to the variance of different processes, and the sensitivity of these indices to parameters differ between these examples. This reflects the different life history strategies of trees and humans, the difference between between age-classified populations with low fertility, and a size-classified population with strongly size-dependent fertility, and the difference between assumptions of Bernoulli or Poisson distributed rewards. Vastly different life histories can be incorporated into the Markov chain with reward framework, allowing for the investigation of life history in many species, and from different perspectives.

## Discussion

Lifetime reproductive output is an outcome of the life cycle. Any demographic model implies a distribution for LRO, just as it implies more familiar measures such as *R*_0_ and life expectancy. Even in a deterministic environment, the LRO is a random variable; the stochasticity arises from two sources: the random pathway that the individual follows through its life and the random fertility it exhibits at each stage on that pathway.

Our results in “Markov chains with rewards as a model for LRO” provide the analytical machinery needed to calculate all the statistical properties of LRO that follow from a specified set of stages, a transition matrix, and the moments of stage-specific fertility. These statistical properties include measures of variability among individuals (the variance, CV, skewness, opportunity for selection, etc.). It is important to recognize that this variability does not reflect heterogeneity, genetic or otherwise. Every individual is subject to the same set of vital rates at any stage in the life cycle. Only the outcomes of applying those rates vary; the variation is thus due to individual stochasticity.

The variance, or standardized variance, calculated from demographic models for a variety of species, is large (see also Caswell 2011; van Daalen and Caswell 2015; Steiner et al. 2010). Individual stochasticity creates a large apparent opportunity for selection that is not, in fact, a true opportunity. In this sense, as emphasized by Steiner and Tuljapurkar (2012), the calculations of individual stochasticity provide a neutral model for LRO. A number of comparisons of calculated variance (due to stochasticity) and observed variance (due to some mix of stochasticity and heterogeneity) have shown that stochasticity may explain a significant amount, or all, of the observed variance (Caswell 2011; Van Daalen and Caswell 2015; Steiner et al. 2010).

It is important to remember what neutral model results do, and do not, imply (Caswell 1976). The results show that a certain amount of variance can be accounted for by stochasticity, and hence, that the mere observation of such variation is no evidence for heterogeneity. It does not prove that the observed variance is stochastic, or that there is no heterogeneity, as pointed out by Steiner and Tuljapurkar (2012). It calls for a comparison with models explicitly incorporating heterogeneity, either observed or unobserved, as suggested by Cam et al. (2016). For examples of this approach, see Caswell (2014); Hartemink et al. (2017); Jenouvrier et al. (2016).

The results of Theorem 1 provide an exact solution to the calculation of the statistics of LRO, including both components (within and between pathways; see “Partitioning variance: within and between pathways”). We also provide a complete sensitivity analysis for LRO. Theorem 2 makes it possible to calculate the sensitivity and elasticity of all the moments of LRO and all the statistics calculated from those moments, with respect to changes in mortality, transition probabilities, and the moments of stage-specific fertility. The results include the sensitivity to changes in mean fertility (holding variance constant) and variance in fertility (holding the mean constant).

The formulae for the moments in Theorem 1 and the sensitivities in Theorem 2 are complicated and opaque, because the relationships between LRO and the life cycle structure, the moments of reproduction, the probabilities of survival, and the infinite diversity of pathways through the life cycle, are complicated. Simplifications that permit qualitative generalities are always welcome, and more work on this will be valuable.

We applied sensitivity analysis to several populations of humans and a population of trees. The patterns of sensitivity and elasticity of LRO that we report for these populations have not been described before. Some suggestive patterns appear; they warrant further investigation.

In long-lived age-classified populations with low reproductive output, as diverse as the nineteenth century Swedes, mid-twentieth century Hutterites, the twenty-first century Dutch, and Hadza and Ache hunter-gatherers, the sensitivity of mean LRO to mortality is negative. Most populations show a positive sensitivity of variance in LRO to mortality for the first 40 years of life, only showing a small negative sensitivity between ages 20 and 40. The Ache and Hadza have more pronounced negative sensitivity around these ages, with the Hadza showing negative sensitivity across the first 40 years of life. The sensitivity of Crow’s $\mathcal {I}$ to mortality in the first 40 years of life is positive, showing that an increase in mortality would increase the apparent opportunity for selection of lifetime reproduction. Patterns for elasticities are similar, though smaller in magnitude.

All populations show broadly similar patterns for the sensitivity of LRO to fertility. The sensitivities of mean LRO and variance in LRO to fertility are positive. The sensitivity of Crow’s $\mathcal {I}$ to fertility is negative. The elasticity of mean and variance to fertility is positive, but the elasticity of Crow’s $\mathcal {I}$ is negative. According to these results, populations in modern countries would reduce the apparent opportunity for selection of LRO with increasing fertilities, and high-fertility populations such as Hutterites and hunter-gatherers would only slightly reduce the opportunity for selection should fertilities increase.

On average, the variance in LRO is 59% between pathways and 41% within pathways. However, the individual populations differ significantly in these contributions by sources of variance. In the twenty-first century in Japan, Sweden, and the Netherlands, only 1.2% of variance in LRO is between pathways and 98.8% is within pathways. Variance in LRO of the high-fertility populations, the Ache, Hadza, and Hutterites, is 71% between pathways and 29% within pathways.

In contrast, in *T. canadensis*, with size-dependent demography, high fertility, and strongly increasing fertility with size, we find that the elasticities of both mean and variance in LRO to mortality are negative across all size classes. The elasticities of these statistical properties to changes in fertility in the reproductive classes are positive. In case of the elasticity to growth transition rates, mean and variance in LRO once again show similar patterns. Elasticity to stasis, i.e., not growing, is negative for the first five size classes, and positive for the last size class.

Lifetime reproductive output interests population ecologists and epidemiologists (for whom *R*_0_ is a measure of population growth), evolutionary biologists (for whom variance in LRO is a measure of potential selection), and human demographers (for whom declines in LRO following the demographic transition pose serious social policy challenges). It is thus important that our analysis is not restricted to any one class of population models. It applies equally to age-structured, stage-structured, and multistate models, and to any reproductive strategy. It also applies to periodic and stochastic time-varying models, by applying Theorems 1 and 2 to the models in Caswell (2011). The method can also be applied to rewards other than reproductive output, including health status (Caswell and Zarulli 2015) and economic transfers (Caswell and Kluge 2015).

The transition matrix **U** is part of any population projection matrix; the Compadre and Comadre matrix databases provide many examples (Salguero-Gómez et al. 2016; Salguero-Gómez et al. 2015). The *mean* reproductive reward matrix **R**_1_ can be obtained from the projection matrix, but the higher moments cannot and require assumptions of a parametric distribution for fertility. We encourage researchers with the appropriate reproduction data to report not only mean fertility but also the higher moments, or even the entire distribution.

Individual stochasticity arises in both reproductive output and in survival or longevity. Our results here complement the analysis of variation in longevity using Markov chain methods, which are widely used in ecology (e.g., Cochran and Ellner 1992, Caswell 2001, 2006, 2009, Horvitz and Tuljapurkar 2008, Tuljapurkar and Horvitz 2006) and have well-developed sensitivity analyses. The Markov chain with reward model now permits a similarly deep analysis of lifetime reproduction and its sensitivity analysis.

## A Derivations

### A.1 Proof of Theorem 1: moments of LRO

We assume a finite state absorbing Markov chain with transition matrix **P** given by Eq. 3, with *τ* transient states, *α* absorbing states, and *s* = *τ* + *α* total states. The (*i*,*j*) entry of the *s* × *s* matrix **R**_*k*_ is the *k*th moment of the reproductive reward associated with the transition from state *j* to state *i*. We assume that no rewards accrue to individuals in absorbing states, so columns *τ* + 1 to *s* of **R**_*k*_ are zero.

The *i*th entry of the *s* × 1 vector ***ρ***_*m*_ is the *m*th moment or remaining lifetime reproductive output for an individual in stage *i*. Caswell (2011, Proposition 1) derived an iterative approximation for the moment vectors,

69 $$\begin{array}{@{}rcl@{}} \boldsymbol{\rho}_{m}(t\!+\!1) \!=\! \sum\limits_{k=0}^{m} \left(\!\!\begin{array}{c} m \\ k \end{array}\!\!\right) \!\left( \mathbf{P} \circ \mathbf{R}_{m-k} \right)^{\textsf{T}} \!\boldsymbol{\rho}_{k}(t) \quad m=1,2,\ldots \end{array} $$

which converges to the lifetime accumulation, for each stage, as *t* →*∞*. To obtain an analytical solution for the moment vectors, we solve (69) for its equilibrium.

The equilibrium moment vector ***ρ***_*m*_ satisfies

70 $$ \boldsymbol{\rho}_{m} = \left( \begin{array}{c} m\\ 0 \end{array}\right) \left( \textbf{P} \circ \textbf{R}_{m} \right)^{\textsf{T}} \boldsymbol{\rho}_0 + \sum\limits_{k=1}^{m-1} \left( \begin{array}{c} m\\ k \end{array}\right)\left( \textbf{P} \circ \textbf{R}_{m} \right)^{\textsf{T}} \boldsymbol{\rho}_{k} + \left( \begin{array}{c} m\\ m \end{array} \right) \left( \textbf{P} \circ \textbf{R}_{0} \right)^{\textsf{T}} \boldsymbol{\rho}_{m}  $$

Because ***ρ***_0_ = **1**_*s*_ and **R**_0_ is a *s* × *s* matrix of ones, this simplifies to

71 $$ \left( \textbf{I} - \textbf{P}^{\textsf{T}} \right) \boldsymbol{\rho}_{m} = \left( \textbf{P} \circ \textbf{R}_{m} \right)^{\textsf{T}} \mathbf{1}_{s} + {\sum}_{k=1}^{m-1} \left( \begin{array}{c} m\\ k \end{array}\right)\left( \textbf{P} \circ \textbf{R}_{m-k} \right)^{\textsf{T}} \boldsymbol{\rho}_{k} .  $$

Solving (71) directly for ***ρ***_*m*_ is impossible because the matrix (**I** −**P** ^T^) is singular. However, since no rewards accumulate in the absorbing states, so we need solve only for the vector of remaining LRO from the transient states, which we denote by $\tilde {\boldsymbol {\rho }}_{m}$. Multiplying both sides of Eq. 70 by the matrix **Z** in Eq. 14 gives

72 $$ \tilde{\boldsymbol{\rho}}_{m} = \textbf{Z} \left( \textbf{P} \circ \textbf{R}_{m} \right)^{\textsf{T}} \mathbf{1}_{s} + \sum\limits_{k=1}^{m-1} \left( \begin{array}{c} m\\ k \end{array}\right)\textbf{Z} \left( \textbf{P} \circ \textbf{R}_{m-k} \right)^{\textsf{T}} \textbf{Z}^{\textsf{T}} \tilde{ \boldsymbol{\rho}}_{k} + \textbf{Z} \textbf{P}^{\textsf{T}} \textbf{Z}^{\textsf{T}} \tilde{\boldsymbol{\rho}}_{m} $$

Noting that

73 $$\begin{array}{@{}rcl@{}} \textbf{Z} \textbf{P}^{\textsf{T}} \textbf{Z}^{\textsf{T}} &=& \textbf{U}^{\textsf{T}} \end{array} $$

74 $$\begin{array}{@{}rcl@{}} \textbf{Z} \left( \textbf{P} \circ \textbf{R}_{m-k} \right)^{\textsf{T}} \textbf{Z}^{\textsf{T}} &=& \left( \textbf{U} \circ \tilde{\textbf{R}}_{m-k} \right)^{\textsf{T}} \end{array} $$

we see that $\tilde {\boldsymbol {\rho }}_{m}$ must satisfy

75 $$ \tilde{\boldsymbol{\rho}}_{m} = \textbf{Z} \left( \textbf{P} \circ \textbf{R}_{m} \right)^{\textsf{T}} \mathbf{1}_{s} + \sum\limits_{k=1}^{m-1} \left( \begin{array}{c} m\\ k \end{array}\right)\left( \textbf{U} \circ \tilde{\textbf{R}}_{m-k} \right)^{\textsf{T}} \tilde{\boldsymbol{\rho}}_{k} + \textbf{U}^{\textsf{T}} \tilde{\boldsymbol{\rho}}_{m}  $$

Solving (75) for $\tilde {\boldsymbol {\rho }}_{m}$ gives (19) and completes the proof of Theorem 1.

### A.2 Proof of Theorem 2: sensitivity analysis of moments of LRO

To derive the sensitivity result of Theorem 2, we will break the result (19) into three types of terms and differentiate each of these in turn. We rewrite (19) as

76 $$ \underbrace{\left( \textbf{N}^{\textsf{T}} \right)^{-1} \tilde{\boldsymbol{\rho}}_{m}}_{A} = \underbrace{\textbf{Z} \left( \textbf{P} \circ \textbf{R}_{m} \right)^{\textsf{T}} \mathbf{1}_{s}}_{B} + \sum\limits_{k=1}^{m-1} \left( \begin{array}{c} m\\ k \end{array}\right) \underbrace{\left( \textbf{U} \circ \tilde{\textbf{R}}_{m-k} \right)^{\textsf{T}} \tilde{\boldsymbol{\rho}}_{k}}_{C}  $$

Equation 76 contains three types of terms:

77 $$\begin{array}{@{}rcl@{}} A &=& \left( \textbf{N}^{\textsf{T}} \right)^{-1} \tilde{\boldsymbol{\rho}} \end{array} $$

78 $$\begin{array}{@{}rcl@{}} B&=&\textbf{Z} \left( \textbf{P} \circ \textbf{R} \right)^{\textsf{T}} \mathbf{1}_{s} \end{array} $$

79 $$\begin{array}{@{}rcl@{}} C &=&\left( \textbf{U} \circ \tilde{\textbf{R}} \right)^{\textsf{T}} \tilde{\boldsymbol\rho} \end{array} $$

We differentiate each of these in turn. Differentiating (77) gives

80 $$ d A = d \left[ \left( \textbf{N}^{\textsf{T}} \right)^{-1}\right] \tilde{\boldsymbol{\rho}} + \left( \textbf{N}^{\textsf{T}} \right)^{-1} d \tilde{\boldsymbol{\rho}} . $$

Substituting

81 $$ \left( \textbf{N}^{\textsf{T}} \right)^{-1} = \textbf{I}_{s} - \textbf{U}^{\textsf{T}} $$

gives

82 $$ d A = - \left( d \textbf{U}^{\textsf{T}} \right) \tilde{\boldsymbol{\rho}} + \left( \textbf{I} - \textbf{U}^{\textsf{T}} \right) d \tilde{\boldsymbol{\rho}} , $$

and applying the vec operator yields

83 $$ d \text{vec} A = - \left( \tilde{\boldsymbol{\rho}}^{\textsf{T}} \otimes \textbf{I} \right) \textbf{K}_{\tau \tau} d \text{vec} \ \textbf{U} + \left( \textbf{I} - \textbf{U}^{\textsf{T}} \right) d \tilde{\boldsymbol{\rho}}  $$

Differentiating term *B* gives

84 $$ d B = \textbf{Z} \left( d \textbf{P} \circ \textbf{R} \right)^{\textsf{T}} \mathbf{1} + \textbf{Z} \left( \textbf{P} \circ d \textbf{R} \right)^{\textsf{T}} \mathbf{1}  $$

Applying the vec operator to both sides of Eq. 84 yields

85 $$ d \text{vec} B = \left( \mathbf{1}^{\textsf{T}} \otimes \textbf{Z} \right) \textbf{K}_{ss} \left[ \mathcal{D}(\text{vec} \ \textbf{R}) d \text{vec} \ \textbf{P} + \mathcal{D} (\text{vec} \ \textbf{P}) d \text{vec} \ \textbf{R} \right]  $$

Differentiating term *C* gives

86 $$ d C = \left( d \textbf{U} \circ \tilde{\textbf{R}} \right)^{\textsf{T}} \tilde{\boldsymbol{\rho}} + \left( \textbf{U} \circ d \tilde{\textbf{R}} \right)^{\textsf{T}} \tilde{\boldsymbol{\rho}} + \left( \textbf{U} \circ \tilde{\textbf{R}} \right) d \tilde{\boldsymbol{\rho}} . $$

Applying the vec operator yields

87 $$\begin{array}{@{}rcl@{}} d \text{vec} \ C &=& \left( \tilde{\boldsymbol{\rho}}^{\textsf{T}} \otimes \textbf{I} \right) \textbf{K}_{\tau \tau} \left[ \mathcal{D}(\text{vec} \ \tilde{\textbf{R}}) d \text{vec} \ \textbf{U} + \mathcal{D}(\text{vec} \ \textbf{U}) d \text{vec} \ \tilde{\textbf{R}} \right] \\&&+ \left( \textbf{U} \circ \tilde{\textbf{R}} \right) d \tilde{\boldsymbol{\rho}} \end{array} $$

Next, differentiate (76) and substitute (83)–(87) where appropriate; this leads to

88 $$\begin{array}{@{}rcl@{}} && \left( \textbf{N}^{\textsf{T}} \right)^{-1} d \tilde{\boldsymbol{\rho}}_{m} = \underbrace{\left( \mathbf{1}_{s}^{\textsf{T}} \otimes \textbf{Z} \right) \textbf{K}_{ss} \left[ \mathcal{D} \left( \text{vec} \ \textbf{R}_{m} \right) d \text{vec} \ \textbf{P} + \mathcal{D}(\text{vec} \ \textbf{P}) d \text{vec} \ \textbf{R}_{m} \right]}_{\textbf{V}_{m}} \\ && + \sum\limits_{k=1}^{m-1} \left( \begin{array}{c} m\\ k \end{array}\right) \underbrace{\left( \tilde{\boldsymbol{\rho}}_{k} \otimes \textbf{I}_{\tau} \right) \textbf{K}_{\tau \tau} \left[ \mathcal{D} \left( \text{vec} \ \tilde{\textbf{R}}_{m-k} \right) d \text{vec} \ \textbf{U} + \mathcal{D}(\text{vec} \ \textbf{U}) d \text{vec} \ \tilde{\textbf{R}}_{m-k} \right] + \left( \textbf{U} \circ \tilde{\textbf{R}}_{m-k}\right)^{\textsf{T}} d \tilde{\boldsymbol{\rho}}_{k}}_{\textbf{W}_{k,m-k}} \\ && + \underbrace{ \left( \tilde{\boldsymbol{\rho}}_{m}^{\textsf{T}} \otimes \textbf{I}_{\tau} \right) \textbf{K}_{\tau \tau} d \text{vec} \ \textbf{U}}_{\textbf{X}_{m}} \end{array} $$

Using the terms **V**_*m*_, **W**_*k*,*m*−*k*_, and **X**_*m*_ identified in Eq. 88, multiplying both sides by **N** ^T^, and replacing differentials with derivatives with respect to a vector ***𝜃*** of parameters give

89 $$ \frac{d \tilde{\boldsymbol{\rho}}_{m}}{d \boldsymbol{\theta}^{\textsf{T}}} = \textbf{N}^{\textsf{T}} \left( \textbf{V}_{m} + \sum\limits_{k=1}^{m-1} \left( \begin{array}{c} m\\ k \end{array}\right)\textbf{W}_{k,m-k} + \textbf{X}_{m} \right)  $$

which completes the derivation of Theorem 2.

The term **V**_*i*_ in Eq. 89 contains the derivative *d*vec **P**/*d* ***𝜃*** ^T^. Because **P** is a block structured matrix and **0** and **I** are constants, the derivative of **P** can be written as a linear combination of the derivatives of **U** and **M**,

90 $$ \frac{d \text{vec} \ \textbf{P}}{d \boldsymbol{\theta}^{\textsf{T}}} = \textbf{C}_{1} {d \text{vec} \ \textbf{U} \over d \boldsymbol{\theta}^{\textsf{T}}} + \textbf{C}_{2} {d \text{vec} \ \textbf{M} \over d \boldsymbol{\theta}^{\textsf{T}}},  $$

where

91 $$\begin{array}{@{}rcl@{}} \textbf{C}_{1} &=& \left( \begin{array}{c} \textbf{I}_{\tau} \\ \mathbf{0}_{\alpha \times \tau} \end{array}\right) \otimes \left( \begin{array}{c} \textbf{I}_{\tau} \\ \mathbf{0}_{\alpha \times \tau} \end{array}\right) \end{array} $$

92 $$\begin{array}{@{}rcl@{}} \textbf{C}_{2} &=& \left( \begin{array}{c} \textbf{I}_{\tau} \\ \mathbf{0}_{\alpha \times \tau} \end{array}\right) \otimes \left( \begin{array}{c} \mathbf{0}_{\tau \times \alpha} \\ \textbf{I}_{\alpha} \end{array}\right) \end{array} $$

(Caswell and van Daalen 2016). In many applications, only a single absorbing state (death) exists, in which case *α* = 1 and Eqs. 91 and 92 simplify to Eq. 35.

### A.3 Sensitivity analysis of the statistics of LRO

The sensitivity analysis of the statistical properties of LRO is obtained by differentiating and applying the vec operator to Eqs. 21–24. Several matrix calculus results are used frequently in these sensitivity calculations. Roth’s theorem, i.e.,

93 $$ \text{vec}\ (\textbf{ABC})=(\textbf{C}^{\textsf{T}} \otimes \textbf{A}) \text{vec} \ \textbf{B}, $$

is applied often, as is the rule for taking the vec of a Hadamard product,

94 $$ \text{vec}\ (\textbf{A} \circ \textbf{B}) = \mathcal{D}(\textbf{A})\text{vec}\ (\textbf{B}) + \mathcal{D}(\textbf{B})\text{vec}\ (\textbf{A}). $$

For notational clarity, we redefine some of the statistical properties of LRO as follows in this section only:

95 $$\begin{array}{@{}rcl@{}} \textbf{v} &=& V(\tilde{\boldsymbol{\rho}}) \end{array} $$

96 $$\begin{array}{@{}rcl@{}}\ \textbf s &=& SD(\tilde{\boldsymbol{\rho}}) \end{array} $$

97 $$\begin{array}{@{}rcl@{}} \textbf{C} &=& CV(\tilde{\boldsymbol{\rho}}) \end{array} $$

98 $$\begin{array}{@{}rcl@{}} \boldsymbol{\mathcal{I}} &=& \mathcal{I}(\tilde{\boldsymbol{\rho}}) \end{array} $$

99 $$\begin{array}{@{}rcl@{}} \textbf D &=& \mathcal{D}(\tilde{\boldsymbol{\rho}}_{1}) \end{array} $$

#### Variance

Differentiating the variance in lifetime reproductive output as given by Eq. 21 gives

100 $$ d \textbf{v}= d\tilde{\boldsymbol{\rho}}_{2} -d(\tilde{\boldsymbol{\rho}}_{1} \circ \tilde{\boldsymbol{\rho}}_{1}). $$

Applying the vec operator results in

101 $$ d \textbf{v}= d\tilde{\boldsymbol{\rho}}_{2} -d \text{vec}\ (\tilde{\boldsymbol{\rho}}_{1} \circ \tilde{\boldsymbol{\rho}}_{1}), $$

which, using the rule for taking the vec of a Hadamard product, yields

102 $$ d \textbf{v}= d\tilde{\boldsymbol{\rho}}_{2} -\textbf{D} d\tilde{\boldsymbol{\rho}}_{1}-\textbf{D} d\tilde{\boldsymbol{\rho}}_{1}. $$

Taking the derivatives with respect to a vector of parameters ***𝜃*** and rearranging give

103 $$ \frac{d \textbf{v}}{d \boldsymbol{\theta}^{\textsf{T}}} = \frac{d \tilde{\boldsymbol{\rho}}_{2}}{d \boldsymbol{\theta}^{\textsf{T}}}-2 \textbf{D} \frac{d \tilde{\boldsymbol{\rho}}_{1}}{d \boldsymbol{\theta}^{\textsf{T}}}, $$

as found in Eq. 38.

#### Standard deviation

To obtain the sensitivity of the standard deviation of LRO, we rewrite (22) as

104 $$ \textbf{s} \circ \textbf{s} = \textbf{v} $$

and differentiate this giving us

105 $$ d (\textbf{s} \circ \textbf{s}) = d \textbf{v}. $$

Using the vec operator, we obtain

106 $$ d \text{vec}\ (\textbf{s} \circ \textbf{s}) = d \textbf{v}, $$

where, after replacing the differentials with derivatives with respect to ***𝜃*** and applying the Hadamard-rule on the left-hand side, we find

107 $$ 2\mathcal{D}\left( \textbf{s}\right)\frac{d \textbf{s}}{d \boldsymbol{\theta}^{\textsf{T}}}= \frac{d \textbf{v}}{d \boldsymbol{\theta}^{\textsf{T}}} $$

Solving for *d* **s**/*d* ***𝜃*** ^T^ yields Eq. 39.

#### Coefficient of variation

The sensitivity of the coefficient of variation is obtained by differentiating

108 $$ \textbf{C} = \textbf{D}^{-1} \textbf{s} $$

from Eq. 23, resulting in

109 $$ d \textbf{C}=d\left( \textbf D^{-1}\right)\textbf{s} +\textbf D^{-1} d\left( \textbf{s}\right) .  $$

However, for any nonsingular matrix **Y**,

110 $$ d(\textbf{Y}^{-1}) = -\textbf{Y}^{-1} d(\textbf Y) \textbf Y^{-1} . $$

Thus, Eq. 109 can be written as

111 $$ d \textbf{C} = -\textbf D^{-1}d\left( \textbf D\right)\textbf D^{-1}\textbf{s} +\textbf D^{-1} d\left( \textbf{s}\right). $$

Applying the vec operator and Roth’s theorem yields

112 $$ d \textbf{C}= -\left( \textbf{s}^{\textsf{T}} \left[\textbf{D}^{-1}\right]^{\textsf{T}} \otimes \textbf{D}^{-1}\right) d \text{vec}\left( \textbf D\right) +\textbf D^{-1} d\left( \textbf{s}\right). $$

Noting that

113 $$ \textbf{s}^{\textsf{T}} \left( \textbf D^{-1}\right)^{\textsf{T}} = \textbf{C}^{\textsf{T}} $$

and that **D** can be rewritten as

114 $$ \textbf{D}=\textbf{I} \circ (\mathbf{1}_{\tau} \tilde{\boldsymbol{\rho}}_{1}^{\textsf{T}}) $$

we obtain

115 $$ d \textbf{C}=-\left( \textbf{C}^{\textsf{T}} \otimes \textbf D^{-1}\right) d \text{vec}\left( \textbf{I} \circ \left[\mathbf{1}_{\tau} \tilde{\boldsymbol{\rho}}_{1}^{\textsf{T}}\right]\right) +\textbf D^{-1} d\left( \textbf{s}\right). $$

Using the rule for taking the vec of a Hadamard product and applying Roth’s theorem once more yields

116 $$ d \textbf{C} =-\left( \textbf{C}^{\textsf{T}} \otimes \textbf D^{-1}\right) \mathcal{D}\left( \text{vec} \ \textbf{I}\right) \left( \textbf{I} \otimes \mathbf{1}_{\tau}\right) d\left( \tilde{\boldsymbol{\rho}}_{1}\right)+\textbf D^{-1} d\left( \textbf{s}\right). $$

Replacing the differentials with derivatives with respect to ***𝜃*** gives

117 $$ \frac{d \textbf{C}}{d \boldsymbol{\theta}^{\textsf{T}}}=-\left( \textbf{C}^{\textsf{T}} \otimes \textbf D^{-1}\right) \mathcal{D} (\text{vec} \ \textbf{I}) (\textbf{I} \otimes \mathbf{1}_{\tau}) \frac{d \tilde{\boldsymbol{\rho}}_{1}}{d \boldsymbol{\theta}^{\textsf{T}}}+\textbf{D}^{-1} \frac{d \textbf{s}}{d \boldsymbol{\theta}^{\textsf{T}}}. $$

#### Crow’s index $\mathcal {I}$

The opportunity for selection, also known as Crow’s index, can be calculated from the coefficient of variation, as shown in Eq. 24. Differentiating this equation results in

118 $$ d \boldsymbol{\mathcal{I}}= d \text{vec}\left( \textbf{C} \circ \textbf{C}\right). $$

Applying the vec operator to the Hadamard product yields

119 $$ d \boldsymbol{\mathcal{I}}= \mathcal{D}(\textbf{C}) d\left( \textbf{C}\right)+\mathcal{D}(\textbf{C}) d\left( \textbf{C}\right). $$

Replacing the differentials with derivatives of Crow’s $\mathcal {I}$ with respect to ***𝜃*** yields

120 $$ \frac{d \boldsymbol{\mathcal{I}}}{d \boldsymbol{\theta}^{\textsf{T}}}= 2\mathcal{D}(\textbf{C})\frac{d \textbf{C}}{d \boldsymbol\theta^{\textsf{T}}} $$

### A.4 Deriving sensitivities for the examples

To obtain the sensitivities for mean LRO and other statistics, the following pieces are required:

121 $$ \frac{d \text{vec} \ \textbf{U}}{d \boldsymbol{\theta}^{\textsf{T}}}, \frac{d \text{vec} \ \textbf{P}}{d \boldsymbol{\theta}^{\textsf{T}}}, \text{ and } \frac{d \text{vec} \ \textbf{R}_{i}}{d \boldsymbol{\theta}^{\textsf{T}}}. $$

In the case of both humans and trees, a single stage of death is incorporated, so that the derivative of **P** with respect to ***𝜃*** can be obtained from the derivative of **U** with respect to ***𝜃***, as shown in Eq. 35. As the transition matrix depends on mortality rates and the reward matrix depends on fertility rates, the required pieces for sensitivity analysis become

122 $$ \frac{d \text{vec} \ \textbf{U}}{d \boldsymbol{\mu}^{\textsf{T}}} \ \text{ and } \frac{d \text{vec} \ \textbf{R}_{i}}{d \textbf{f}^{\textsf{T}}}. $$

The way these matrices depend on the underlying vectors of parameters differs between species, as shown below for humans and trees.

#### Humans

In humans, **U** is a function of the mortality rates ***μ*** through its survival probabilities ***σ***. **U** depends on ***σ*** as

123 $$ \textbf{U} = \textbf{Y} \circ (\mathbf{1} \boldsymbol{\sigma}^{\textsf{T}}) , $$

where **Y** is a *τ* × *τ* matrix with ones on the subdiagonal and zeros elsewhere. Given that

124 $$ \boldsymbol{\sigma} = e^{-\boldsymbol{\mu}} , $$

the derivative of ***σ*** becomes

125 $$ d \boldsymbol{\sigma} =- \mathcal{D}(\boldsymbol{\sigma})d \boldsymbol{\mu}.$$

As such, the complete analytical expression for the sensitivity of the transition matrix to the mortality schedule is

126 $$ \frac{d \text{vec} \ \textbf{U}}{d \boldsymbol{\mu}^{\textsf{T}}} = - \mathcal{D}(\text{vec}\ \textbf{Y})(\textbf{I} \otimes \mathbf{1})\mathcal{D}(\boldsymbol{\sigma}). $$

The sensitivity of the moments of **R** to a vector of fertility rates depends on the kind of reproductive strategy used. In the case of humans, fertility rate is the *chance* of producing a single offspring. With the subsequent assumption that the moments of the reward matrix follow a Bernoulli distribution,

127 $$ \frac{d \textbf{R}_{1}}{d \textbf{f}^{\textsf{T}}} = \frac{d \textbf{R}_{2}}{d \textbf{f}^{\textsf{T}}} =\frac{d \textbf{R}_{3}}{d \textbf{f}^{\textsf{T}}} $$

Therefore, we only need to derive the sensitivity of **R**_1_ to **f** to know all the moments of the reward matrix. Equation 11 can be rewritten as

128 $$ \textbf{R}_{1} = \mathbf{1}_{s} \textbf{f}^{\textsf{T}} \textbf{Z} . $$

Differentiating this entails taking the derivative on both sides and applying Roth’s theorem. Then, the equation for the sensitivity is simply

129 $$ \frac{d \textbf{R}_{1}}{d \textbf{f}^{\textsf{T}}} = \left( \textbf{Z}^{\textsf{T}} \otimes \textbf{1}_{s} \right) .  $$

#### Trees

In trees, the transition matrix consists not only of survival rates but also growth/stasis/regression rates (see Equation 43). By differentiating (43) with respect to ***Σ***, we obtain

130 $$ \frac{d \text{vec} \ \textbf{U}}{d \text{vec}^{\textsf{T}} \boldsymbol{\Sigma}} = (\textbf{I}^{\textsf{T}} \otimes \textbf{G}).$$

As **U** now depends on mortality rate through ***Σ***, the sensitivity of the transition matrix to ***μ*** is

131 $$ \frac{d \text{vec} \ \textbf{U}}{d \boldsymbol{\mu}^{\textsf{T}}} = \frac{d \text{vec} \ \textbf{U}}{d \text{vec}^{\textsf{T}} \boldsymbol{\Sigma}} \frac{d \text{vec} \boldsymbol{\Sigma}}{d \boldsymbol{\mu}^{\textsf{T}}}.$$

Given that ***Σ*** can be written as ***Σ*** = **I** ∘ (**1** ***σ*** ^T^), differentiating, applying the vec operator, using the rule for taking the vec of a Hadamard product, and applying Roth’s theorem give

132 $$ \frac{d \text{vec} \boldsymbol{\Sigma}}{d \boldsymbol{\sigma}^{\textsf{T}}} = \mathcal{D}(\text{vec} \ \textbf{I}) (\textbf{I}^{\textsf{T}} \otimes \mathbf{1}). $$

Combining Eqs. 130 and 132 with the derivative of ***σ*** to ***μ*** derived in Eqs. 125 and 131 becomes

133 $$ \frac{d \text{vec} \ \textbf{U}}{d \boldsymbol{\mu}^{\textsf{T}}} = - (\textbf{I}^{\textsf{T}} \otimes \textbf{G})\mathcal{D}(\text{vec} \ \textbf{I}) (\textbf{I}^{\textsf{T}} \otimes \mathbf{1})\mathcal{D}(\boldsymbol{\sigma}) . $$

The sensitivity of the transition matrix to the growth matrix is found by differentiating (43) with respect to **G**

134 $$ \frac{d \text{vec} \ \textbf{U}}{d \text{vec}^{\textsf{T}} \textbf{G}} = (\boldsymbol{\Sigma}^{\textsf{T}} \otimes \textbf{I}). $$

However, a complication arises from the fact that **G** is column-stochastic, that is, the probabilities of staying or moving between stages always sums to 1. Sensitivity analysis works on the premise of a small change in the underlying parameters effecting a chance in the model outcome. In the case of **G**, a small additive change would result in the loss of the column-stochastic property. Fortunately, we can instead use a proportional change. Caswell (2001, 2013) presents a method of proportionally compensating for a perturbation of a single entry in a column-stochastic matrix by proportionally subtracting the perturbation from the other entries in that column. In matrix notation, this becomes

135 $$ \frac{d \text{vec} \ \textbf{U}}{d \text{vec}^{\textsf{T}} \boldsymbol{\Theta}} = \frac{d \text{vec} \ \textbf{U}}{d \text{vec}^{\textsf{T}} \textbf{G}} \frac{d \text{vec} \ \textbf{G}}{d \text{vec}^{\textsf{T}} \boldsymbol{\Theta}} $$

where

136 $$\begin{array}{@{}rcl@{}} \frac{d \text{vec} \ \textbf{G}}{d \text{vec}^{\textsf{T}} \boldsymbol{\Theta}} = \textbf{I}_{s^2} - \sum\limits_{i=1}^{s} \left( \textbf{E}_{ii} \otimes \mathcal{D} \left[\textbf{G}(:,i)\right] \textbf{C} \mathcal{D} \left[\mathbf{1} - \textbf{G}(:,i)\right]^{-1}\right), \\\text{ with} \quad \textbf{C}=\textbf{E} - \textbf{I}. \end{array} $$

In trees, reproduction is assumed to adhere to a Poisson distribution as opposed to a Bernoulli distribution. Higher moments of the reward matrix therefore depend on **R**_1_ as follows:

137 $$\begin{array}{@{}rcl@{}} \textbf{R}_{2} &=& \textbf{R}_{1} + \textbf{R}_{1} \circ \textbf{R}_{1} \ \text{and} \end{array} $$

138 $$\begin{array}{@{}rcl@{}} \textbf{R}_{3} &=& \textbf{R}_{1} + 3 \textbf{R}_{1} \circ \textbf{R}_{1} + \textbf{R}_{1} \circ \textbf{R}_{1} \circ \textbf{R}_{1} . \end{array} $$

By differentiating the expression for **R**_2_ we obtain

139 $$ d \textbf{R}_{2} = d\left[\textbf{R}_{1}\right] + d\left[\textbf{R}_{1} \circ \textbf{R}_{1}\right]. $$

Applying the vec operator yields

140 $$ d \text{vec} \ \textbf{R}_{2} = d \text{vec} \ \textbf{R}_{1} + 2 \mathcal{D}(\text{vec} \ \textbf{R}_{1})d \text{vec} \ \textbf{R}_{1}. $$

Replacing the differentials with the derivative of **R**_2_ with respect to **R**_1_ gives

141 $$ \frac{d \text{vec} \ \textbf{R}_{2}}{d \text{vec} \ \textbf{R}_{1}} = \textbf{I}_{s^2}+ 2 \mathcal{D}(\text{vec} \ \textbf{R}_{1}). $$

Then, the derivative of **R**_2_ with respect to **f** becomes

142 $$ \frac{d \text{vec} \ \textbf{R}_{2}}{d \textbf{f}^{\textsf{T}}} = \frac{d \text{vec} \ \textbf{R}_{2}}{d \text{vec} \ \textbf{R}_{1}}\frac{d \text{vec} \ \textbf{R}_{1}}{d \textbf{f}^{\textsf{T}}}, $$

which, using the expression above and recalling (129), becomes

143 $$ \frac{d \text{vec} \ \textbf{R}_{2}}{d \textbf{f}^{\textsf{T}}} = \left( \textbf{I}_{s^2}+ 2 \mathcal{D}(\text{vec} \ \textbf{R}_{1})\right)(\textbf{Z}^{\textsf{T}} \otimes \mathbf{1}_{s}). $$

Similarly, we can obtain the sensitivity of **R**_3_ to **f** by differentiating (138), resulting in

144 $$ d \textbf{R}_{3} = d \textbf{R}_{1} + 3 d\left( \textbf{R}_{1} \circ \textbf{R}_{1}\right) + d \left( \textbf{R}_{1} \circ \textbf{R}_{1} \circ \textbf{R}_{1} \right). $$

Applying the vec operator then gives

145 $$\begin{array}{@{}rcl@{}} d \text{vec} \ \textbf{R}_{3} &=& d \text{vec} \ \textbf{R}_{1} + 6 \mathcal{D}(\text{vec} \ \textbf{R}_{1})d \text{vec} \ \textbf{R}_{1}\\ &&+ 3 \mathcal{D} \left( \text{vec} (\textbf{R}_{1} \circ \textbf{R}_{1}) \right)d \text{vec} \ \textbf{R}_{1}, \end{array} $$

where the differentials can be replaced with derivatives of **R**_3_ with respect to **R**_1_, yielding

146 $$ \frac{d \text{vec} \ \textbf{R}_{3}}{d \text{vec} \ \textbf{R}_{1}} = \textbf{I}_{s^2}+ 6 \mathcal{D}(\text{vec} \ \textbf{R}_{1}) + 3 \mathcal{D} \left( \text{vec} (\textbf{R}_{1} \circ \textbf{R}_{1}) \right). $$

Given that

147 $$ \frac{d \text{vec} \ \textbf{R}_{3}}{d \textbf{f}^{\textsf{T}}} = \frac{d \text{vec} \ \textbf{R}_{3}}{d \text{vec} \ \textbf{R}_{1}}{d \text{vec} \ \textbf{R}_{1} \over d \textbf{f}^{\textsf{T}}}, $$

and that the derivative of **R**_1_ with respect to **f** is (129), the sensitivity of **R**_3_ to **f** is

148 $$ \frac{d \text{vec} \ \textbf{R}_{3}}{d \textbf{f}^{\textsf{T}}} =\left[\textbf{I}_{s^2}+ 6 \mathcal{D}(\text{vec} \ \textbf{R}_{1}) + 3 \mathcal{D} \left( \text{vec} (\textbf{R}_{1} \circ \textbf{R}_{1}) \right)\right](\textbf{Z}^{\textsf{T}} \otimes \mathbf{1}_{s}) . $$

## Footnotes

1 This is a slightly more general concept than lifetime reproductive success (Clutton-Brock 1988) because it can accommodate many different operational definitions of reproduction, as are often encountered in ecological and demographic analysis.

2 Tuljapurkar et al. (2009) independently and at the same time introduced the term “dynamic heterogeneity” to refer to the same random variation . We continue to use individual stochasticity because it more accurately describes the process creating the inter-individual variance and allows us to distinguish heterogeneity that is static from that which changes over the life of an individual.

3 If the Markov chain is ergodic, or if it is absorbing but the absorbing states continue to collect rewards, then rewards will continue to accumulate forever. Lifetime accumulated rewards in such cases converge only if a discount factor is imposed, making delayed rewards less valuable than immediate rewards. We will present results for ergodic chains elsewhere.
